# Crop resilience *via* inter-plant spacing brings to the fore the productive ideotype

**DOI:** 10.3389/fpls.2022.934359

**Published:** 2022-09-21

**Authors:** Ioannis Tokatlidis

**Affiliations:** Department of Molecular Biology and Genetics, Democritus University of Thrace, Alexandroupolis, Greece

**Keywords:** absence of compensation, density dependence, honeycomb breeding, low-input agriculture, optimum density, plant yield efficiency, resource use efficiency, yield gap

## Abstract

Natural selection favors the competitive ideotype, enabling native plants to survive in the face of intense competition. The productive ideotype is the goal of artificial selection to achieve high crop yields *via* the efficient use of resources in a self-competition regime. When breeding is established under inter-genotypic competition, the competitive ideotype dominates and may fictitiously become selectable. The productive ideotype becomes selectable at the nil-competition regime, where widely spaced individuals prevent plant-to-plant interference for any input. Principal reasons bring to the fore the productive ideotype that combines low competitiveness and improved plant yield efficiency. Crop spacing *via* the productive ideotype is mandated to alleviate the varying optimum density and ensure efficient use of resources inter-seasonally, cope with intra-field variation and optimize resource use, compensate for missing plants and promote stability, counteract unpredictable stresses and offer a buffer against environmental diversity, and adopt low-input agriculture to conserve natural resources and the environment. For breeding toward the productive ideotype, nil-competition is the due condition to overcome the confounding effects of competition, maximize phenotypic differentiation and facilitate selection from an early segregating generation, optimize heritability due to moderated environmental variance and experimental designs that sample spatial heterogeneity, apply high selection pressure focusing exclusively on the targeted genotype, and avoid the risk of bias selection or loss of desired genotypes due to proximity to empty hills. The view of a modern crop variety composed of genotype(s) belonging to the productive ideotype is a viable option to reach crop resilience serving sustainability in enormously fluctuating agroecosystems.

## Introduction

Because of climate change, agriculture faces enormously fluctuating conditions accompanied by unpredictable abiotic stresses (Mirás-Avalos and Baveye, 2018). Agriculture is vulnerable to the risk and impacts of weather events incident upon global climate change, resulting in more variable crop yields (Lavalle et al., 2009). In days to come, environmental diversity would affect plant growth and crop yield by elevated atmospheric CO_2_ concentration, higher temperatures, and altered precipitation regimes (Altieri et al., 2015). Extreme weather events, like heat, drought, heavy storms, and frost, might be more frequent and intensive in the future (Altieri et al., 2015), while heat and drought have already become severe even in northern European countries (Brás et al., 2021). Flexible and resilient crops are imperative, and adaptable to continuous environmental change to maintain the ability to farm and produce food in the future (Lichtfouse et al., 2009).

**Figure 1:**
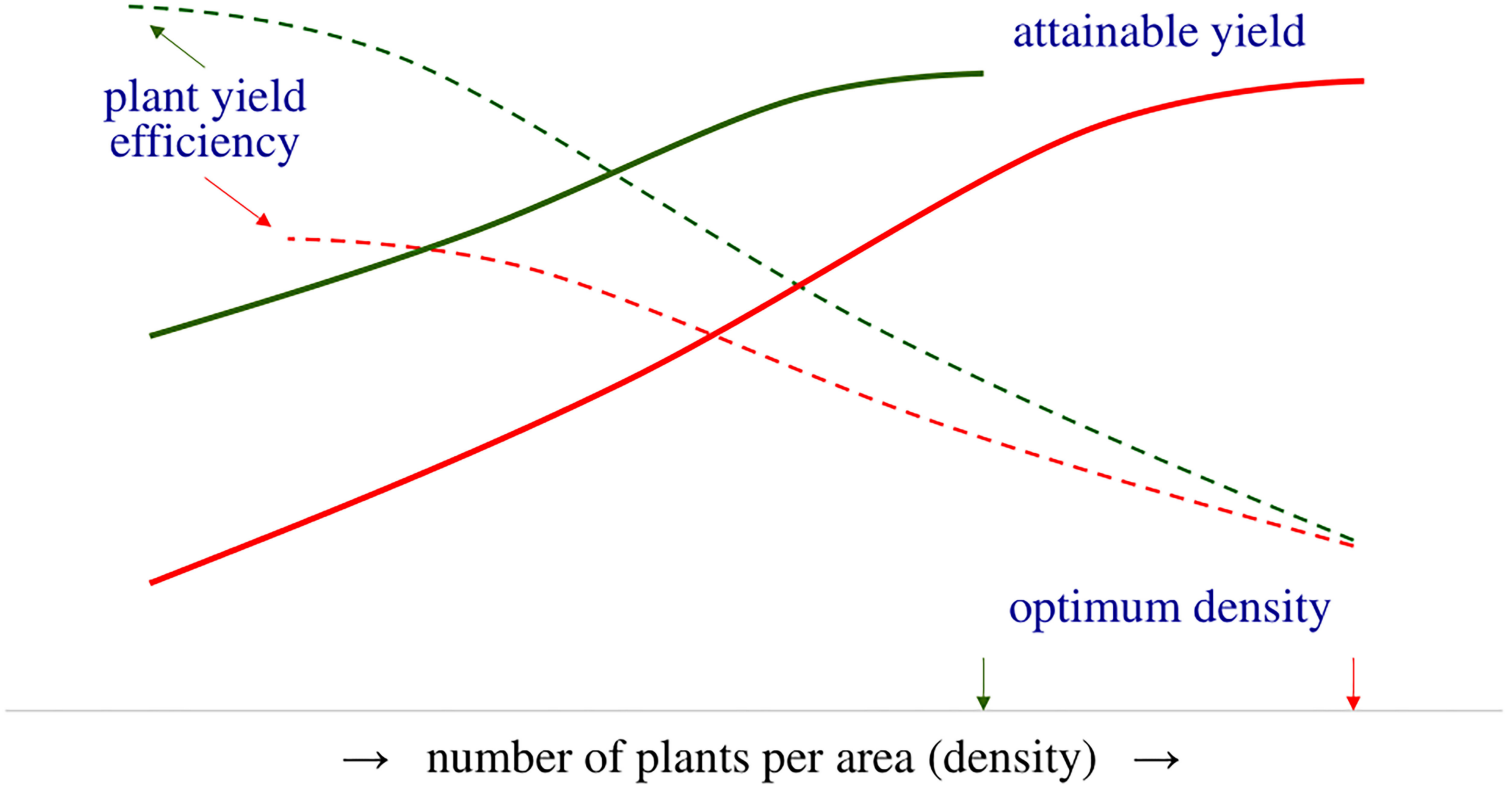
Plant yield efficiency (dotted lines) is a determining factor in crop yield (solid lines) and is critical to achieving attainable yield. Varieties short of plant yield efficiency (red lines) require high (optimum) density to achieve the attainable yield, while improved plant yield efficiency reduces the optimum density.

In this study, an agroecosystem is defined as a cultivated ecosystem corresponding to the spatial unit of a crop. The accomplished crop (farming) yield is the product of the mean grain yield per plant and the number of finally established plants in the field (Friedman, 2016). The mean grain yield per plant depends on the ability of the single plant to respond to resources, defined as plant yield efficiency (Tokatlidis, 2017). Thus, plant yield efficiency is a crucial factor in achieving the attainable potential yield (Figure 1), i.e., the highest possible farming yield depending on the availability of resources and the prevalent soil and climatic conditions. Attainable yield varies across agroecosystems and is rarely accomplished due to the multiplicity of the implicated factors, e.g., variety coupled with farmer skillfulness and crop management; thus, actual farming yield lags behind the attainable yield resulting in a yield gap (Van Ittersum et al., 2013; Anderson et al., 2016; O'Brien et al., 2021). Numerous studies have shown the existence of a considerable yield gap in staple crops, such as wheat (*Triticum* spp. L.; Lollato et al., 2019), maize (*Zea mays* L.; Rufo et al., 2015), rice (*Oryza sativa* L.; Yang et al., 2008), soybean [*Glycine max* (L.) Merr.] (Egli and Hatfield, 2014), beans (*Phaseolus vulgaris* L.; Eash et al., 2019), and other pulses (Gireesh et al., 2019). The consensus is that crops are not able to utilize natural and additional inputs. According to Van Ittersum et al. (2013), the yield gap is caused by an unclear combination of factors which include limiting factors (e.g., nutrients) and reducing factors (e.g., pest and diseases, soil compaction).

Therefore, approaches should be adopted to substantially increase crop yields over the coming decades to keep pace with global food demand (Van Ittersum et al., 2013). Crop improvement can advance the level of attainable yields and, in consequence, actual yields (Senapati and Semenov, 2020). Also, narrowing the yield gap is a mandate to reach food adequacy and security in the days ahead. Several studies have made an effort to investigate the contributing factors and ways of resolving such crises. For example, Fischer et al. (2009) proposed agronomic management and breeding to lessen the yield gap. Van Ittersum et al. (2013) recommended optimal crop management with regard to tillage, sowing, fertilization, density, and crop protection to attain the attainable yield. O'Brien et al. (2021) suggested genetic, environmental, and management options as strategies to increase food security.

This study focuses on two variety characteristics as potential contributors to the yield gap in grain-producing crops, both revolving around the plant population density (hereafter “density”). The competitive ability of the variety's component genotype is critical to the level of the intra-field variation, responsible for interplant inequality, uneven use of resources, and yield loss (Tokatlidis, 2017). Erratic optimum density is another substantial contributor to yield loss, determined by the ability of the single plant to respond to abundant resources, i.e., plant yield efficiency (Tokatlidis, 2013, 2014). The “productive ideotype” instead of the “competitive ideotype” is described as the ideal constituent genotype of a modern variety. The productive ideotype, combining low competitive ability and high plant yield efficiency, is a prerequisite for crop spacing, i.e., reduced crop density per area; crop spacing instead of increased crop density (crowding) is essential to mitigate the interplant inequality, alleviate the uncertainty of optimum density, and achieve efficient use of resources. The absence of competition is an inviolable condition in crop breeding pursuing the productive ideotype.

## Productivity vs. competitiveness

### Inter-genotypic competition in crop breeding

Competition refers to the negative effects on plant growth caused by neighboring plants, which usually occurs by reducing the availability of resources. In a natural ecosystem, plants battle to survive under competition between both different species (inter-specific) and different genotypes of the same species (inter-genotypic); thus, natural selection acts on the requirement of competitiveness (Donald, 1963). At the other edge, in a common homogeneous agroecosystem, competition exists between genetically identical plants (intra-genotypic). Overall, grain yield per area (crop yield) is the ultimate goal, and breeding focuses on resource use efficiency and productivity (Donald, 1968). Breeders should consider the vital difference between the natural ecosystem and agroecosystem to determine the ideal variety and the conditions to look for it (Papadakis, 1940a).

In crop breeding, potential competition between plants under consideration for selection belongs to the inter-genotypic type. Papadakis paid attention to the per plant (genotype) available space 90 years ago (Papadakis, 1935a, 1937a,b, 1940a,b, 1941). He introduced the evaluation of widely spaced single plants in crop breeding to eliminate the inter-genotypic competition and obtain wheat varieties that replaced all other varieties in Greece for almost 20 years (Papadakis, 1982). Donald also emphasized inter-genotypic competition and postulated that a successful crop ideotype would be a weak competitor, proposing the selection of non-competitive or communal ideotypes (Donald, 1963, 1968, 1981; Donald and Hamblin, 1976). Fasoulas (1973, 1981, 1987) considered the confounding effects of inter-genotypic competition in identifying superior genotypes and defined two distinct competition regimes (Fasoulas, 1988, 1993; Fasoula, 1990; Fasoula and Fasoula, 1997). In mixtures of genotypes, such as segregating progeny lines, inter-genotypic competition reflects the allo-competition regime. In the common homogeneous crop stand, competition among genetically identical plants imitates the self-competition regime (intra-genotypic competition). In the allo-competition regime, productive genotypes suffocate due to the presence of competitive ones. While the productive genotype is the breeding target for cultivation in a state of self-competition, it may be unrecognizable under allo-competition. As inter-genotypic competition whittles away, i.e., the distance between individual plants progressively increases, productive genotypes gradually express their yielding capacity; they achieve full expression when they escape the obstructive influence of competitive genotypes (Papadakis, 1937a; Chatzoglou and Tokatlidis, 2012; Ninou et al., 2014). A widespread consensus exists about an inverse connection of the genotype's ability to perform with its competitiveness (Papadakis, 1940a; Donald, 1963; Fasoulas, 1988, 1993; Sedgley, 1991; Fasoula and Fasoula, 1997; Pan et al., 2003; Fischer and Rebetzke, 2018). In other words, productive genotypes outperform competitive genotypes when individual plants are wide apart to prevent plant-to-plant interference for any input and intra-specific competition ceases to exist, i.e., the regime defined by Fasoulas “nil-competition” (Fasoulas, 1988, 1993; Fasoula, 1990; Fasoula and Fasoula, 1997).

Consequently, two extreme ideotypes arise. On the one hand, the strong competitor benefits when it develops in competition with other genotypes but may not perform well on its own; it is the “competitive ideotype” that resembles the genotype preferred by natural selection. On the other edge, the weak competitor suffers from inter-genotypic competition but stands out for yielding performance when develops alone; is the “productive ideotype” (Tokatlidis, 2017).

### The concept of intra-field variation

A dense stand is a resource-limited regime where underground and aboveground resources are insufficient to satisfy each plant's needs, e.g., space, water, nutrients, and light. According to Schwinning and Weiner (1998), larger plants often obtain a disproportionate share of the contested resources and suppress the growth of their smaller neighboring plants, in a phenomenon called size-asymmetric competition. Size-asymmetric competition precipitates developmental dissimilarity, which interferes with the equal share of inputs (Tokatlidis, 2017). Plants with a competitive advantage consume more resources than their share, while their neighbors are required to utilize less than their share (Fasoula and Fasoula, 1997; Fasoula and Tokatlidis, 2012). The degree of plant-to-plant variability reflects the intensiveness and implications of interplant inequality (Tokatlidis, 2017).

Genetic and acquired differences are the sources of interplant inequality. Genetic heterogeneity of the allo-competition regime is an obvious and inevitable cause of developmental dissimilarities. Acquired inequality comes from any non-genetic factor that causes plant-to-plant variability and is present under both allo- and self-competition. Pre-emergence factors are the delay and uneven plant emergence due to differences in sowing depth, insects, birds, rodents, herbicide residues (Pommel and Bonhomme, 1998), soil pathogens, seed vigor, soil temperature (Hamman et al., 2002), seed and seedling characteristics, seedbed components, plant density (Lamichhane et al., 2018), and soil crusting (Laker and Nortjé, 2019). Post-emergence contributors are age differences and spatial heterogeneity concerning soil, light interception, nutrition, diseases, weeds, and pests (Pan et al., 2003). The competitive genotype can enhance any occasional advantage for its benefit over neighboring plants that lag in establishment, growth, and development (Schwinning and Weiner, 1998; Benjamin, 2017; Tokatlidis, 2017).

The growth of a plant and the plant size variability are directly related to the number, size, and proximity to neighbors. A greater degree of size inequality reflects variation in relative growth rates induced by intra-specific competition. In most experiments, including varying plant density, plant-to-plant variability increased with density; the change in the coefficient of variation (CV) for growth rate can determine the role of intra-specific competition in generating intra-field variation (Benjamin, 2017). Relevant investigations indicated a positive correlation of density with CV per plant for grain yield and other agronomic traits in various crops, e.g., barley (*Hordeum vulgare* L.; Hamblin et al., 1978), rye (*Secale cereale* L.; Kyriakou and Fasoulas, 1985), and numerous in maize (Tollenaar and Wu, 1999; Echarte et al., 2000; Tokatlidis et al., 2005, 2010a; Liu and Tollenaar, 2009). Increased density enhances plant-to-plant variability since early vegetative stages because the most suppressed individuals of the stand intercept less radiation per unit leaf area than the dominant ones (Rossini et al., 2016). Hence, increased interplant inequality at higher densities is a direct manifestation of enhanced intra-specific competition.

Several researchers have recognized the importance of intra-specific competition that according to Adler et al. (2018) may be stronger than inter-specific competition. The consequent interplant inequality causes reduced resource use and yields because yield gains of bigger plants do not offset yield losses of smaller plants (Joernsgaard and Halmoe, 2002). Stafford et al. (1996) highlighted the necessity to overcome the intra-field variation and optimize resource use in cereal crops. High intra-specific competition pressure in maize promotes intra-field variation and the appearance of extreme plant hierarchies with different abilities to capture scarce resources (Mayer et al., 2012). Sunflower yield is negatively associated with responsiveness to intra-specific competition (Sadras et al., 2000). Intensified intra-specific competition results in losses of dry mass accumulation and grain yield (Mondo et al., 2013). Besides environmental conditions, crowding, and management practices, the level of acquired interplant inequality is a matter of the variety's genetic background (Fasoula and Tokatlidis, 2012). Therefore, seeking a type of variety that performs well at low densities and at the same time, withstanding factors inducing acquired intra-field variation is an insightful pursuit (Tokatlidis, 2017).

### The concept of density dependence

**Figure 2:**
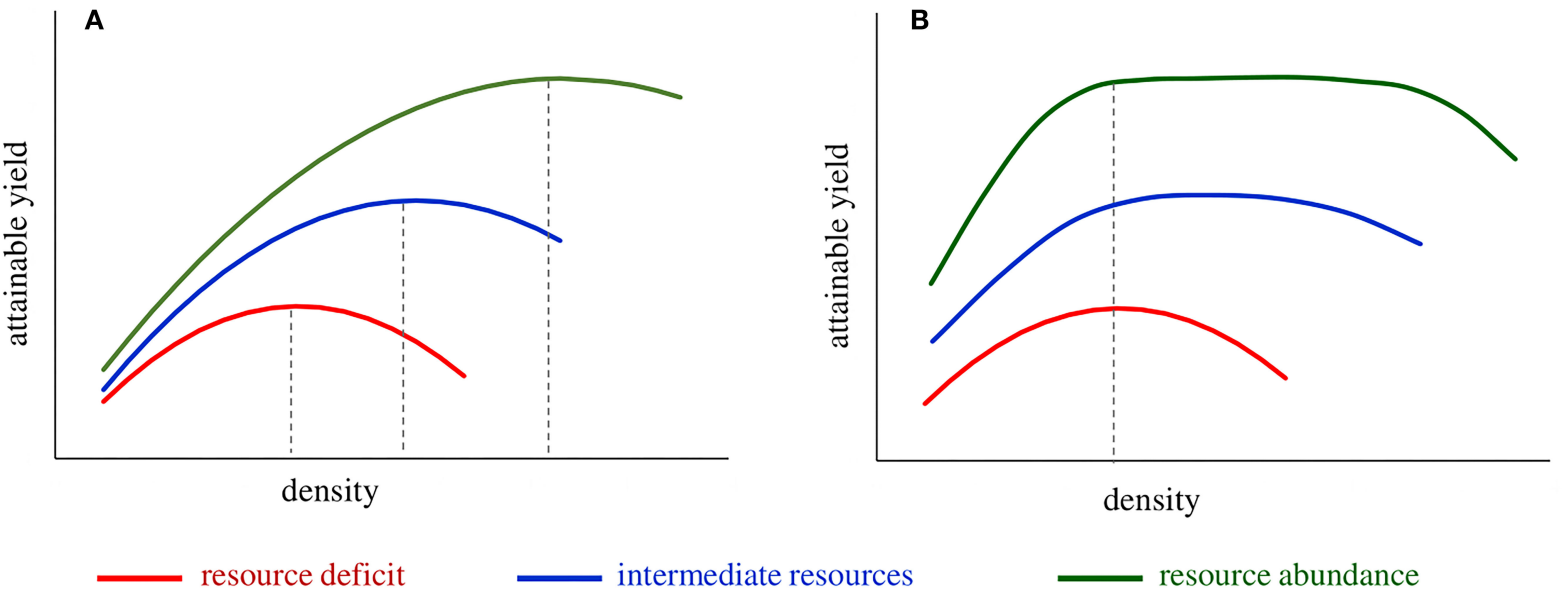
The theoretical performance of the **(A)** density-dependent variety falling short in plant yield efficiency and **(B)** density-independent variety that comprises the productive ideotype of high plant yield efficiency. The density-dependent variety exhibits erratic optimum density across environments, i.e., low under resource deficit conditions (e.g., dry season) and increasing as growing conditions improve; failure at sowing to predict and establish the most appropriate population density results in yield penalty. The density-independent variety exhibits a similar performance under resource deficit conditions (low and narrow range of optimum density); however, the range of optimum density widens as resources increase. Crop spacing *via* density-independent varieties is a viable option to ensure crop efficiency in resource use inter-seasonally and bridge the yield gap, thanks to the ability to take advantage of abundant resources at the single-plant level.

Plant breeding and agronomic practices to serve intensive agriculture have unconsciously focused mainly on tolerance to high densities and not on plant yield efficiency. Indicatively in maize, during the hybrid era, a progressive grain yield increase was followed by a parallel rise in the density (Mansfield and Mumm, 2014). Differences in grain yield between old and recently released hybrid varieties are more a function of density rather than plant yield potential (Ciampitti and Vyn, 2014; Assefa et al., 2018; Gonzalez et al., 2018). Below-optimum seeding rates in wheat may reduce resource use efficiency, yield, and final profit depending on the level of resource availability (Tokatlidis, 2014; Fischer et al., 2019; Lollato et al., 2019; Bastos et al., 2020). Transition to higher densities if accompanied by plant stagnation in yielding capacity results in varieties exhibiting typically high and usually erratic optimum density, i.e., the density-dependent variety (Figure 2A). Density dependence poses a threat to sustainability due to the destructive effect of high density under intense stress conditions (e.g., drought) and the difficulty at the sowing time to predict the optimum density (Tokatlidis et al., 2001; Berzsenyi and Tokatlidis, 2012; Tokatlidis, 2013, 2014, 2017).

Previous studies have analyzed the adverse effects of density dependence on grain yield stability concerning maize (Tokatlidis and Koutroubas, 2004; Tokatlidis et al., 2011a, 2015; Berzsenyi and Tokatlidis, 2012; Tokatlidis, 2013; Mylonas et al., 2020) and wheat (Tokatlidis et al., 2006; Tokatlidis, 2014). Improvement of plant yield potential, i.e., the crop's ability to produce grain at the single-plant level, is the solution to managing density dependence (Fasoulas, 1993; Fasoula and Fasoula, 2000, 2002; Duvick, 2005; Fasoula and Tokatlidis, 2012; Tokatlidis, 2017). The genotype plant yield potential reflects its plant yield efficiency and yielding capacity per se (Tokatlidis et al., 2015; Tokatlidis, 2017). As density declines and the distance between neighboring plants increases, more inputs are available for each plant. Hence, improved plant yield efficiency implies that individuals are highly responsive to additional inputs and increase their grain yield (Figure 1). Improved plant yield efficiency reduces the lower limit of optimum density, and coupled with tolerance to density, contributes to an extended range of optimum density, describing the density-independent variety (Figure 2B). Density-independent varieties would allow crop spacing, i.e., lower than currently used densities, of prime importance in terms of stability demonstrated below.

### The productive ideotype

The concepts of intra-field variation and density dependence mirror the productive ideotype rather than the competitive one as ideal for a modern variety. The productive ideotype combines two component traits, low (inter-specific) competitive ability with high plant yield efficiency:


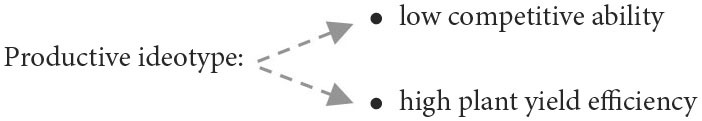


The first component trait is valuable for the variety to evade acquired interplant dissimilarities and promote equality, while the second allows crop spacing without compromising grain yield per area. Key reasons render the productive ideotype the necessary component of the density-independent variety. Expected benefits and the way to look for the density-independent variety are discussed below.

## Benefits from the productive ideotype

### Alleviating the erratic optimum density to promote inter-seasonal stability

A crucial parameter to be addressed is the problem of varying optimum density (Bastos et al., 2020). The issue primarily concerns rainfed crops and greatly affects irrigated crops and arises from the complex variety by density interaction that usually matches the parabolic pattern (Amelong et al., 2017; Tokatlidis, 2017). While the optimum density might be low to accomplish the attainable yield in stressful seasons, the increased attainable yield of favorable seasons is accompanied by increased optimum density (Figure 2A). In other words, the optimum density of the same variety differs among agroecosystems. Due to erratic optimum density, farmers are more likely to fail to establish the most appropriate population, sustaining a yield penalty. The concept has been thoroughly analyzed for maize and wheat in two review articles (Tokatlidis, 2013, 2014), and two extreme examples of potential yield loss are given below.

Across 11 seasons (1989–1999) in a single location (Martonvásár, Hungary), the optimum density of the maize hybrid “Norma” ranged from 5 up to 10 plants/m^2^ (Berzsenyi and Tokatlidis, 2012). The hybrid exhibited the highest and lowest optimum density to reach the yield plateau of 8.9 and 1.9 t/ha for the seasons of 1989 and 1990, respectively. Complete yield loss would occur for the dry season of 1990 if the established density would be high enough to match the optimum of the previous season. This study stimulated a review article early in 2012 (Tokatlidis, 2013), pointing out that such a disaster is likely to happen in a dry season due to a very high density. The prediction of crop disaster came true the following summer in Iowa, USA; in 2018 also, a record drought across Germany caused crop failures. An extreme example also comes from the wheat study in northern Syria (Anderson, 1986), indicating that the potential yield loss of the variety “Buckbuck” in a dry season would be almost 80% if cultivated at the high optimum density of the following more favorable season (Tokatlidis, 2014). Density dependence explains why in arid environments with year-to-year variation in rainfall, farmers often use risk management tactics, such as low plant density, and limit investment in inputs that may be unprofitable in the event of a drought (Eash et al., 2019).

The remedy relies on improved plant yield efficiency, essential to lower and stabilize the optimum density. High plant yield efficiency would convert the density-dependent type of variety to the density-independent one. The density-independent variety could be grown in low populations, the demand of dry seasons, but at the same time could achieve high attainable yields of favorable seasons, thanks to the ability to exploit abundant water and other resources at the single-plant level (Duvick, 2005; Tokatlidis, 2013, 2014; Mylonas et al., 2020). Drought-induced agricultural loss is one of the most costly impacts of extreme weather, and without mitigation, climate change is likely to increase the severity and frequency of future droughts (Glotter and Elliott, 2016). Drought-related cereal production annual losses intensify by more than 3% (Brás et al., 2021). Future agriculture adaptation challenges are not only linked to changes in the long-term average climate but particularly to changing weather extremes and interannual fluctuations (Brás et al., 2021).

### Mitigation of the intra-field variation to enhance resource use efficiency

Great attention has been paid to the inter-field (over-location and/or over-season) variation, however, the intra-field variation is also meaningful. Differences between neighboring plants are prevalent in the field, and unbalanced input use decreases possible profit (Pan et al., 2003; Fasoula and Tollenaar, 2005; Tokatlidis, 2017; Fasoula et al., 2020). A widespread consensus exists that crop yield declines with increasing plant-to-plant variability (e.g., Stafford et al., 1996; Joernsgaard and Halmoe, 2002; Zhai et al., 2018; Shao et al., 2019). The inverse relationship between the level of interplant variation in yield with mean yield is more likely to be exponential, i.e., the Taylor's Power Law (Döring et al., 2015). Even a small increase in plant-to-plant variability from the optimum (lowest) levels may result in substantial yield loss (Tollenaar and Wu, 1999; Tokatlidis and Remountakis, 2020; Pankou et al., in press). In pursuing a minimum plant-to-plant variability, the variety is a determinant besides crop management practices.

Several plants of advanced growth may occur at an early stage, and this initial variation can be the basis for further intra-field variation because of competitive interactions between neighbors (Benjamin, 2017). The two extreme varieties, comprising the competitive or the productive ideotype, will differ in relative performance. The strong competitor will consume inputs insatiably at the expense of neighbors, widening thus its superiority and inducing extra acquired interplant differences. Larger plants benefit from a larger share of resources and frustrate the growth of their smaller neighbors (Schwinning and Weiner, 1998). Inequality intensifies on the presupposition that, at an early stage, the bigger plant has a (genetic) competitive advantage (Tokatlidis, 2017). Such a variety would be prone to intra-field variation. At the other extreme, the weak competitor will slightly take advantage to steal inputs from neighboring plants; thus, it will induce relatively mild acquired intra-field variation. Plants of the weak competitor ideotype in the crop community compete to a minimum degree (Donald, 1963, 1968). Varieties comprising the weak competitor would withstand environmental forces responsible for acquired interplant differences, ensuring, by comparison, an equality regime to optimize the use of resources at the crop level. Genetically determined differences in growth and developmental traits, environmental conditions, and adaptive responses govern interplant inequality (Brabencová et al., 2017).

### Compensation for missing plants to reduce yield loss

Usually, the occasion of missing plants within the crop stand is inevitable. Even if sowing of seed lot with excellent germination capacity is applied, part of it fails to emerge. Lamichhane et al. (2018) reported significant seedling emergence variability of seven field crops both within and among the years attributed to abiotic and biotic stresses. Variation in seedling emergence is an insurmountable obstacle in determining an optimal seeding rate to obtain the optimum density (Tokatlidis, 2014). Post-emergence following stresses increase further the rate of missing plants. In addition, the situation worsens when targeting high densities because the ratio of surviving plants vs. seeds sown declines drastically as the seeding rate increases due to greater intra-specific competition (Spink et al., 2000; Wood et al., 2003; Anderson et al., 2004; Whaley et al., 2004).

Suppose a variety falls short of yield capacity at the single-plant level. In that case, it will be prone to yield loss. Plants neighboring empty hills that cannot exploit the additional inputs will poorly compensate for missing plants, and the overall crop yield will substantially decrease. Results from Pommel and Bonhomme (1998) in maize showed the rate of yield loss to almost parallel the rate of missing plants (Tokatlidis and Koutroubas, 2004); this finding is clear evidence of stagnation of plant yield efficiency, which is why farmers often resow the crop. On the other extreme, a density-independent variety of high plant yield efficiency will withstand yield penalty; individual plants will increase their yield to compensate for missing neighbors.

### Development of multi-genotypic varieties to counteract destructive stresses

Reduced diversity of agricultural systems is due to mono-crop systems to maximize yields under favorable conditions; these systems may lack resilience when faced with changing climate (Isbell, 2015). Severe stress can be entirely destructive for a mono-genotypic variety if the component genotype is susceptible. A literature survey supports the type of multi-genotypic variety as a means of stability. For example, one or more genotypes within the landrace population will yield satisfactorily regardless of the plant's varying biotic and abiotic stress (Zeven, 2002). Similarly, the usefulness of mixtures (multiline varieties and variety mixtures) for disease management has been well demonstrated (Mundt, 2002; Ohtsuki and Sasaki, 2006; Kristoffersen et al., 2020). Hence, the multi-genotypic variety may deserve even more room in agriculture to counteract unpredictable biotic and abiotic stresses (Kristoffersen et al., 2020).

The multi-genotypic variety will be widely adopted if it comprises several compatible genotypes of the productive ideotype, similar in traits like plant height, seed color, and flowering and maturity time. For two reasons, an added value arises if surviving genotypes belong to the productive ideotype, i.e., weak competitor—high plant yield efficiency. First, to mitigate the intra-field variation and ensure equality among plants in the input share. Second, to take advantage of additional inputs resulting from the loss of partners and compensate for them. Such a multi-genotypic variety would offer a buffer against environmental diversity.

### Expansion of low-input agriculture to conserve natural resources and environment

In recent decades, soil degradation, defined as lowering and losing soil functions, fertility, and biodiversity, is becoming more and more serious worldwide and poses a threat to agricultural production and the terrestrial ecosystem (Lal, 2003; Maximillian et al., 2019). Expansion of arable land in developing countries partly offsets the respective decline in developed countries. Natural causal factors intensifying due to climate change, coupled with human activity, i.e., intensive agriculture and chemical inputs, lead to soil degradation in developed countries (Maximillian et al., 2019). Therefore, agriculture faces the challenge of producing more food on ever-shrinking land.

A reason to expand the adoption of low-input agriculture is to continue cultivating degraded soils, and more importantly, to protect natural resources preventing further degradation. Because low-input agriculture is applicable at low densities, developing varieties of high plant yield efficiency is imperative to overcome lower yields. Indicative is the innovative low-input “System of Crop Intensification”, adoption of which increases in Asian, African, and Latin American countries (Adhikari et al., 2018); crop spacing is among the five stable rules to minimize intra-specific competition, giving each plant more room to grow above and below ground, and emphasize the per plant available inputs (Abraham et al., 2014; Uphoff et al., 2015). Crop spacing was proved necessary to enable each plant to attain close to its maximum genetic potential, improve the resource use efficiency of the crop, and increase grain yields initially in rice and later on in maize, wheat, and other crops (Adhikari et al., 2018).

### Additional benefits

In addition to the above-mentioned main reasons, side benefits also arise from growing density-independent varieties and crop spacing, most pertinent to the yield gap. First, crop spacing mitigates the level of plant-to-plant variability and improves crop stand uniformity. Planting at low populations reduces self-shading and increases light absorption by the canopy (Olsen and Weiner, 2007). Cropping at low densities along with a uniform sowing pattern and optimized spatial arrangement also increases harvest index (Fischer and Kertesz, 1976; Siddique et al., 1984; Fang et al., 2010), seed weight, test weight, leaf size, and elongates the grain filling period (Hansen et al., 2005). In maize, low populations improve the synchronization of pollen and silk emergence, thus improving kernel set and reducing the proportion of barren plants (Hashemi-Dezfouli and Herbert, 1992; Tokatlidis and Koutroubas, 2004). Reduced pollen-to-silking interval and increased harvest index, reflecting the partitioning of assimilates to the ear and grain, have been suggested as indicators of tolerance to drought (Duvick, 2005; Lopes et al., 2011). Crops grown at low populations are less susceptible to damage due to frost (Whaley et al., 2004), diseases (Jurke and Fernando, 2008; Farias et al., 2019; Omer et al., 2021), and lodging (Jurke and Fernando, 2008; Chauhan et al., 2021). The potential offset from the reduced population of lower seed cost is remarkable because seed represents one of the essential economic inputs (Spink et al., 2000); reasonably minimized seed waste might be significant concerning food adequacy and security in the future (Tokatlidis, 2014). Overall, the productive ideotype releases the crop from high densities, renders it less variable, and damps down the problem of the yield gap.

## Breeding for the productive ideotype

**Figure 3:**
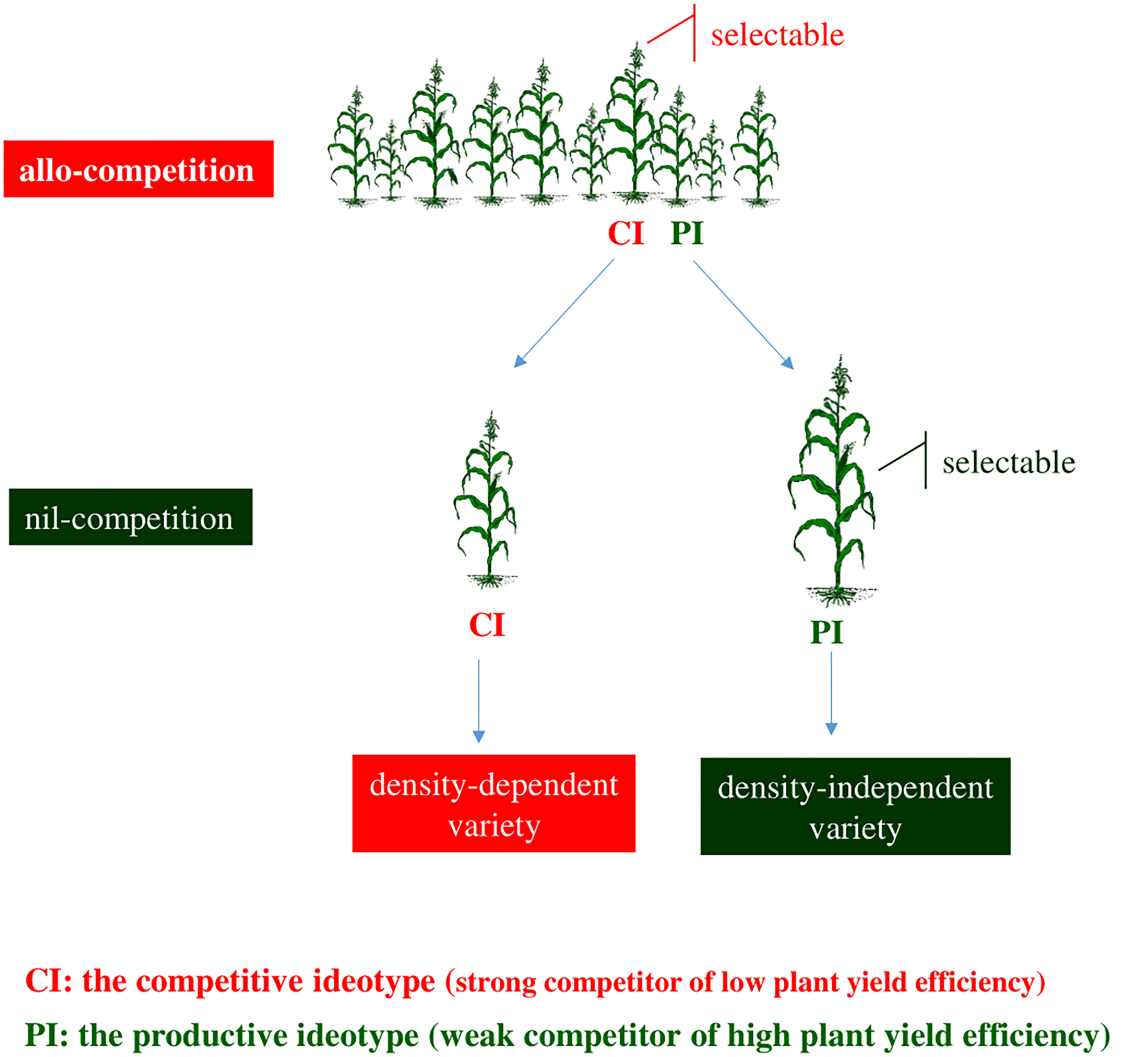
Allo-competition does not correlate to nil-competition. In the allo-competition regime, the competitive ideotype becomes selectable because dominates over the productive ideotype. The productive ideotype becomes selectable at nil-competition where escapes the suspending effects of competition. Breeding at allo-competition leads to varieties that comprise component genotype(s) of low plant yield efficiency exhibiting thus density dependence. Breeding at the nil-competition regime favors component genotype(s) of high plant yield efficiency and density-independent varieties.

In the allo-competition regime, inter-genotypic competition favors plants of the competitive ideotype at the expense of plants of the productive ideotype (Figure 3). Selection within heterogeneous progeny lines leads to density-dependent varieties including genotype(s) of the competitive ideotype. Due to the inverse connection of productivity with competitive ability, genotypes of the productive ideotype are not recognizable under allo-competition conditions. Nil-competition allows plants to express their yielding capacity and facilitates the identification of genotypes of the productive ideotype.

**Figure 4:**
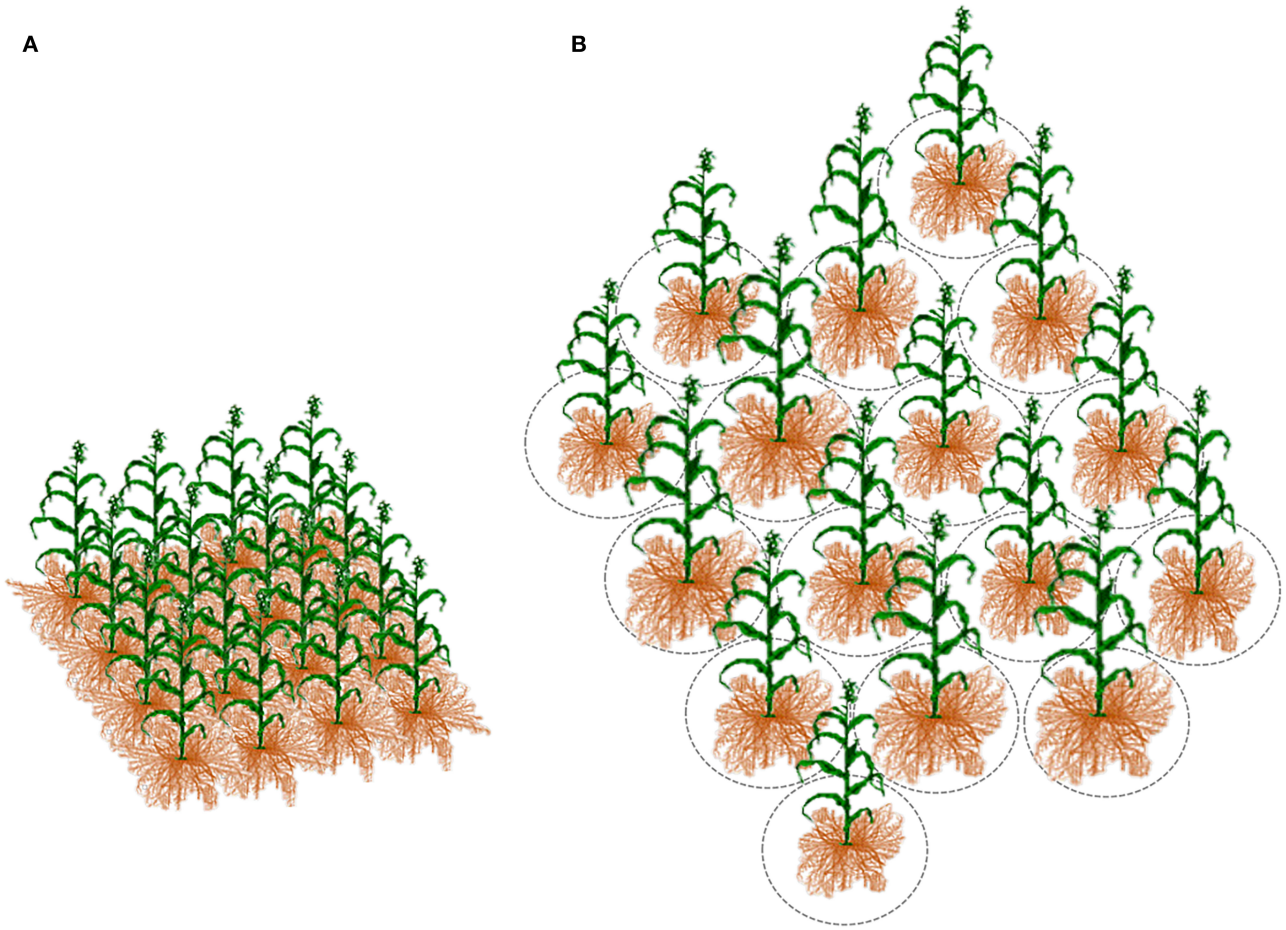
When a progeny line is tested **(A)** at the densely planted plot, the allo-competition regime affects the individual plant growth. In contrast, **(B)** the increased inter-plant distance at the nil-competition regime prevents plant-to-plant interference for any input; critical is the space that corresponds to each plant, delimited by the imaginary circles, which must be the minimum to allow each plant to grow unaffected by the neighboring plants.

**Figure 5:**
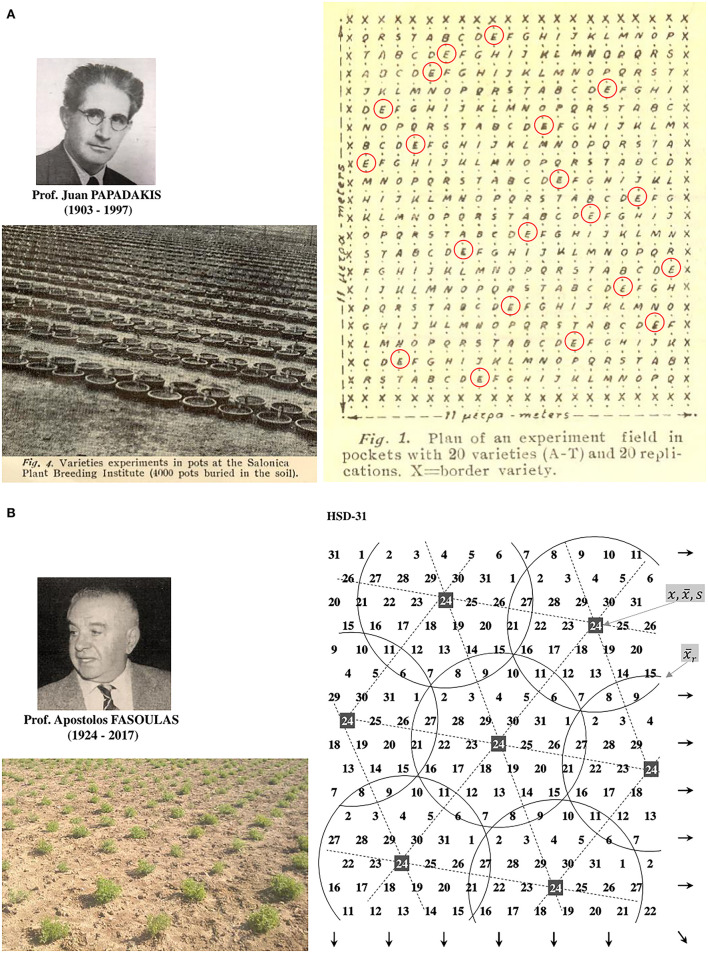
**(A)** The Papadakis' ideas about pots (left) or spaced individual hills substitution for plots, and distribution of entries across the experiment (right), evolved into Fasoula's Method. **(B)** The Fasoulas' Honeycomb Breeding Method includes selection among individual plants at nil-competition (left, a lentil trial) in the configuration of even and systematic entry distribution (right, e.g., the design of 31 entries adapted from Fasoulas and Fasoula, 1995). The plant's yield (*x*) is divided by the mean of the complete circular block (${\bar{x}}_{r}$), resulting in the plant yield index to consider the genotype for possible selection. For a particular entry, e.g., 24, mean yield ($\bar{x}$) or pooled plant yield index reflects the entry's plant yield efficiency. Entry mean divided by its standard deviation (s) is used as a prognostic measure of intra-field inequality (it should be considered cautiously in early segregating generations).

Breeders typically use the plot as an experimental unit to evaluate progeny lines per area (Figure 4A). It seems reasonable to assume the plot mimics the field conditions; however, because of genetic heterogeneity within the plot, the breeding trial is too far to simulate the crop's actual state, especially at early segregating generations. First, it is the inverse connection of the genotype's ability to perform with its competitiveness, elaborated in the concept of inter-genotypic competition. The inter-genotypic competition means that high-yielding plants are competitive and do not necessarily achieve a high crop yield in a pure stand (Weiner et al., 2017; Fischer, 2020). Reynolds et al. (1994) found the yield potential of wheat to be associated with a less competitive ideotype. Therefore, testing heterogeneous early-generation sibling lines at densities corresponding to farming conditions appears senseless (Tokatlidis, 2017). Response to resource availability will typically vary among diverse genotypes to alter genotype ranking (Rebetzke et al., 2014). Another critical factor for replacing the plot as the breeding evaluation unit is the little seed in early generations, preventing large progeny plots for unbiased yield determination. Pooled progeny gives a mixture of genotypes and yields results that may be difficult to interpret, as is the subsequent selection for pure line development and testing (Fischer, 2020). Each plant may represent a unique genotype and deserves due attention, especially in highly heterogeneous early generations. Papadakis (1935a,b) paved the way for replacing the plot, proposing line evaluation in single-plant pots buried in the soil or hills at a great distance, and scattered in the experimental area (Figure 5A). Then Fasoulas (1981, 1988, 1993) went even further by limiting the evaluation unit strictly to the individual plant at nil-competition (Figures 4B, 5B).

### The nil-competition regime

The breeder has two options: establishing the breeding trial and applying selection in either allo-competition or nil-competition conditions (Figure 4). Due to two obstacles, the allo-competition regime is disadvantaged in the objective pursuit of the productive ideotype. First, it increases environmental variance, thus, reducing heritability because of a pronounced acquired interplant variation. The second obstacle is the inter-genotypic competition that disfavors the productive ideotype. The nil-competition regime removes the two obstacles (Fasoula et al., 2020).

Definition of the nil-competition regime by Fasoulas (1981, 1988, 1993) refers to widely spaced individual plants to preclude interference with each other for any input, like space, light, water, and nutrients (Figure 4B). Each plant grows seamlessly and unhindered exploits the available inputs to express its genetic background. The space share is decisive and should be large enough to allow autonomous plant development, both under- and above-ground. Fasoulas (1981, 1988, 1993) developed the “Honeycomb Breeding Method” for crop breeding, in which nil-competition is the first and inviolable principle (Fasoula and Tokatlidis, 2012; Fasoula, 2013). He also constructed the “Honeycomb Selection Designs”, where each plant lies in the center of a circle surrounded by six equidistant plants (Fasoulas, 1988; Fasoulas and Zaragotas, 1990; Fasoulas and Fasoula, 1995). In principle, the procedure has three key characteristics: (1) the absence of inter-genotypic competition to allow recognition of genotypes of the productive ideotype within a progeny line, (2) an equal share of plenty of inputs for each progeny line, and (3) comparable conditions to evaluate and select progeny lines and individual plants objectively. Previous documents have explained the method in detail (Fasoulas, 1988, 1993; Fasoulas and Fasoula, 1995; Fasoula and Fasoula, 1997, 2000, 2002; Fasoula and Tokatlidis, 2012; Fasoula, 2013). Points concerning the purpose of this study are summarized below.

### Response to selection at nil-competition

According to the breeder's equation (Falconer, 1989), theoretically, response to selection is highest under situations of most increased phenotypic differentiation, heritability, and selection pressure. Breeding at nil-competition fully satisfies the three requirements of the breeder's equation. Maximum phenotypic differentiation facilitates the detection of superior genotypes. The negative relationship between competitive and yielding ability ceases to be essential, the environmental impact is blunted, and appropriate designs manage spatial heterogeneity to improve heritability. High selection intensity is applicable, thankfully to the objectiveness of the conditions. Indicative is the study by Kyriakou and Fasoulas (1985), referred to below as “the reference study”. They applied mass selection within a rye population, including 2,000 plants under either allo-competition or nil-competition. Their results are commented on gradually.

#### Nil-competition maximizes phenotypic differentiation

Early-generation selection for grain yield itself is frustrated by the small amounts of seed available (Fischer and Rebetzke, 2018). Single-plant yield increases drastically in response to declining density reaching a plateau at a very low density (Friedman, 2016). Therefore, at nil-competition, each plant has the maximum grain yield, providing plenty of seed for extensive progeny evaluation. Differences between individual plants also widen and reach maximum, thus expediting single-plant selection even from an early segregating generation. Indeed, in the reference study (Kyriakou and Fasoulas, 1985), compared to allo-competition, nil-competition achieved eight times higher mean yield and five times higher standard deviation value. In Fasoula (1990), seven wheat genotypes averaged 52 times higher grain yield per plant and nine times greater differences at nil-competition compared to the typical dense stand. When density decreases, enlarged differences among entries accompany enlarged phenotypic expression (Fasoulas, 1988; Fasoula and Fasoula, 1997, 2000; Tokatlidis et al., 2010a).

#### Nil-competition optimizes heritability

Differential genotype response to resource availability in the allo-competition regime reduces heritability for growth-related traits (Rebetzke et al., 2014). Resolving the confounding effects of inter-genotypic competition, reduced environmental influences on genotype expression, combined with an experimental configuration that samples the spatial heterogeneity, make the nil-competition ideal condition to optimize heritability.

There is a consensus that usually lower densities moderate the environmental variance that arises from the negative relationship between density and CV for single-plant grain yield and other agronomic traits (Rosielle and Hamblin, 1981; Maddonni and Otegui, 2004; Fasoula and Tollenaar, 2005; Tokatlidis et al., 2005, 2010a; Mansfield and Mumm, 2014; Rossini et al., 2016). Thus, nil-competition minimizes the environmental variance unless too much interplant distance increases the occupied land space and the concomitant soil heterogeneity (Kotzamanidis et al., 2009; Tokatlidis et al., 2010a). In the reference study (Kyriakou and Fasoulas, 1985), the 40% lower CV at nil-competition reflected a lower environmental impact on genotype expression.

The honeycomb selection designs have been deployed for dealing with spatial heterogeneity (Fasoulas, 1988; Fasoulas and Zaragotas, 1990; Fasoulas and Fasoula, 1995). A standardized even and systematic entry layout instead of the randomized configuration and implementing the main principles met in other models, such as blocking, replication, and nearest neighbor adjustment on the same baseline, make this experimental model advantageous over the popular ones in reducing the experimental error (Tokatlidis, 2016). It is appropriate to give a simple example. In the initial source population, to consider a particular plant for possible selection, the plant is located in the center of a circle. The absolute yield of the plant is adjusted in relation to the mean value of all the plants included in the circle (of flexible size), resulting in the plant yield index. Thus, all plants are considered for selection on the mean of a moving circular block, simulating the nearest-neighbor adjustment method. Suppose that 31 plants are selected, each constituting a separate progeny line. Any line is evenly distributed across the entire area, thus forming the most comparable condition to decide which one deserves further consideration (Figure 5B). Lines are evaluated on the overall plant yield index to estimate their plant yield efficiency. The inverse value of the single-plant coefficient of variation, named line stability index, qualifies the line's ability to withstand environmentally induced acquired inequality. However, this criterion should be taken into consideration cautiously in early segregating generations due to genetic heterogeneity (Tokatlidis, 2017); in addition, the unconditional use of the stability index may end in bias line evaluation due to a mathematical rather than an agronomically meaningful mechanism of association of the coefficient of variation with mean yield (Döring et al., 2015; Smutná and Tokatlidis, 2021; Pankou et al., in press). Considering the within-line selection, plants that belong to the same progeny line are constantly surrounded by plants of the rest progeny lines, forming the complete systematic circular block. Thus, a fixed block evenly scattered forms the most comparable condition to decide which plant of the selected line will go on the procedure. These principles apply regardless of the number of entries included in the trial.

#### Nil-competition allows the application of high selection pressure

Devoid of fallacious implications of competitive advantages and disadvantages, nil competition ensures that the application of high selection intensity will keep only favorable genotypes. Genotypes of the productive ideotype accumulate at the right edge of the yield distribution of plants grown at nil competition. By targeting the utmost productive genotypes, a breeder can apply high selection pressure. In contrast, at allo-competition conditions, the risk of selecting strong competitors instead of high-yielding genotypes is enhanced with high selection pressure. In the reference study (Kyriakou and Fasoulas, 1985), with increasing the selection pressure, a positive response to selection was obtained in the nil-competition regime, while the reverse was true under competition.

### Avoidance of bias selection or loss of selectable genotypes

In addition to Falconer's requirements, nil-competition utilizes all the genetic variability established in the experiment. In field experiments, missing hills are common. Even slight competition may cause a biased selection or loss of desired genotypes at the dense stand due to empty hills. Under competition, plants neighboring empty hills gain an acquired advantage and may become by error selectable. Thus, considering them for selection may induce bias. In another option, the breeder may discard all the plants surrounding an empty hill. However, omitting them might imply a loss of desired genotypes. Mind that at the rate of 5% empty hills at scattered positions, the surrounding plants (potentially discarded) are at least 35%. Instead, at nil-competition, missing hills do not affect the development and seed production of the neighbors. All the plants, including those adjoining unoccupied hills, can be considered for selection without the risk of bias, and avoid the loss of superior genotypes because of their proximity to unoccupied hills.

## Discussion

Interplant distance, intra-specific competition, and interplant inequality are distinct but closely related issues. The first determines the per area density, i.e., the level of crowding. Intra-specific competition within a crop stand refers to the reduced availability of growth resources due to the presence of neighbors and the inference between plants to consume the limited inputs. Interplant inequality regards the plant developmental dissimilarity (intra-field variation) and unequal input consumption. Crowding accentuates the intra-specific competition, which in turn intensifies the intra-field variation and interplant inequality. Several researchers have recognized the importance of lightening the severity of intra-specific competition to improve crop performance (e.g., Stafford et al., 1996; Sadras et al., 2000; Joernsgaard and Halmoe, 2002; Mondo et al., 2013; Zhai et al., 2018; Shao et al., 2019).

Plant yield efficiency and tolerance to high density determine the lower and upper limits of optimum plant density. If tolerance to density is not accompanied by improved plant yield efficiency, the transition to higher densities results in a narrow spectrum of optimum density and density dependence (Duvick, 2005; Fasoula et al., 2020; Zhang et al., 2021). Due to density dependence, optimum density is inconsistent, particularly in rainfed crops, a crucial obstacle in accomplishing optimal production (Duvick, 2005; Mylonas et al., 2020); thus, potential yield loss due to erratic optimum density may reach the level of crop failure (Tokatlidis, 2013).

Addressing the intra-field variation and density dependence appears imperative. Regardless of the mono- or multi-genotypic type, future varieties should comprise the weak competitor ideotype to withstand and narrow the environmentally induced acquired intra-field variation (Tokatlidis, 2017). If breeders ignore the antagonism between competitivity and productivity, they cannot create productive varieties (Papadakis, 1940a, 1982). It is possible and compatible to bring varieties for reduced intra-field variation, provided the barrier of inter-genotypic competition is overcome (Fasoulas, 1988; Fasoula et al., 2020). The prospect of reduced intra-field variation is offered by the productive ideotype that combines low competitive ability with high yield per se. The productive ideotype, distinguished for plant yield efficiency, drops the bottom limit of optimum density rendering the variety density-independent. The density-independent variety (Figure 2B) offers the prospect of crop spacing, i.e., continuous cultivation in the low density required for dry conditions. The availability of density-independent varieties would tackle the crop uncertainty due to varying optimum densities (Tokatlidis, 2013, 2014; Mylonas et al., 2020). Tokatlidis et al. (1998, 2001, 2005) described a first breeding cycle to approximate density-independent maize hybrids. Fischer et al. (2019) and Fischer (2020) demonstrated that the goal of density-independent variety in wheat is feasible. With regards to weeds, a compensatory mechanism is indispensable because weeds have, by nature, strong genetic competitive abilities. Crop competitiveness against weeds in cereals was associated with a high overall leaf area, an increased number of tillers, and a faster rate of canopy development (Van der Meulen and Chauhan, 2017). Walsh (2019) found crucial crop stand uniformity in minimizing the ongoing impact of weeds. Gaba et al. (2018) attributed reduced weed biomass production to the crop-wheat competitive advantage to take up *N*. The proposed “productive ideotype” competes against weeds *via* early seed germination, an extensive root system, and the ability to capture resources for rapid and vigorous plant growth (Fasoula and Tokatlidis, 2012).

The loss of high-quality land, the slowing in annual yield increases of major cereals, the expanding fertilizer use, and the effect on the environment indicate that we need to develop new strategies to raise grain yields with less impact on the environment (Chapagain and Good, 2015). Crop spacing *via* the density-independent variety may extend the low-input agriculture to conserve natural resources and the environment in replacement of intensive agriculture where it is needed. Low-input cropping systems aim to produce more for the same land area while reducing dependency on external inputs, conserving resources, and reducing negative environmental impacts (Wezel et al., 2015).

Another requirement met by the density-independent variety is to tackle yield loss due to missing plants in both mono- and multi-genotypic varieties. The percentage of missing plants that a density-independent variety could tolerate is a function of optimum density's bottom and upper limits. Supposing missing plants occur at scattered positions, a density proportionately higher than the bottom and up to the upper limit would offer compensation satisfactorily. For example, in the study of Berzsenyi and Tokatlidis (2012), the maize hybrid “Maraton” simulated the density-independent variety exhibiting an optimum density between 6.5 and 9.5 plants/m^2^. Thus, it could tolerate a rate of up to 30% missing plants at 8.5 plants/m^2^. Even wider was the range of optimum density Tokatlidis et al. (2001) reported in maize hybrids developed based on the “productive ideotype” (Tokatlidis, 2013).

Papadakis (1940a, 1982) stated that if breeders ignore the antagonism between competitivity and productivity, they cannot create productive varieties. Nil-competition is the necessary condition for breeding toward the productive ideotype. The honeycomb breeding method removes the confounding effects of competition in recognition of the targeted genotypes. Significant advantages are the per plant plenty of seed production for extensive progeny testing and accentuated phenotypic differentiation enabling early generation single-plant selection. Mitigating the environmental variance and sampling the spatial heterogeneity, the technique optimizes heritability. High selection intensity focusing exclusively on plant yield efficiency is feasible. All the desired genotypes of the surviving genetic material in the breeding trial are potentially selectable without the risk of bias. The approach opens up new, possibly more efficient strategies for early generation selection for potential yield and greater yield progress per unit cost; it also brings the possibility of using remote sensing and introducing new indirect yield selection criteria (Fischer, 2020).

Honeycomb breeding and nil-competition are “inextricably” linked and “integral” issues. Although the term “honeycomb” refers to experimental designs, the primary and inviolable principle that must be strictly adhered to is the absence of competition. Studies in wheat by Mitchell et al. (1982) and Lungu et al. (1987) applied the method at low densities but insufficient to meet the absolute absence of competition, possibly facing the masking effect of competition and the risk of bias; despite promising results, the methodology was rated too complex and labor-intensive. Fischer (2020) attributed the lack of widespread adoption to low heritability due to error variance and a larger area of land needed. On the other hand, Tokatlidis (2016) and Kargiotidou et al. (2016) indicated that breeders do not need to be concerned with the soil heterogeneity that might induce the extra surface required, owing to the even and systematic entry allocation of the honeycomb designs. Discouraging results came from the mass selection at nil-competition in the rye plant (Pasini and Bos, 1990; Bussemakers and Bos, 1999). On the contrary, the method was successful in improving grain yield in several crops, e.g., maize (Tokatlidis et al., 1998, 2001), cotton (*Gossypium hirsutum* L.; Batzios et al., 2001), rice (Ntanos and Roupakias, 2001), bean (Tokatlidis et al., 2010b), and lentil (*Lens culinaris* Medikus; Kargiotidou et al., 2014; Vlachostergios et al., 2018). Honeycomb breeding at nil-competition also succeeded in improving biomass yield in populations of *Dactylis glomerata* L. and *Agropyron cristatum* (L.) Gaertn (Abraham and Fasoulas, 2001) and switchgrass (*Panicum virgatum* L.; Missaoui et al., 2005). Other reports of successful selection exist in snap bean (*Phaseolus vulgaris* L.; Traka-Mavrona et al., 2000), tomato (*Solanum lycopersicum* L.; Christakis and Fasoulas, 2002; Avdikos et al., 2021), and barley (Tsivelikas et al., 2022). Nil-competition, accentuating the interplant phenotypic differences, allowed the method to exploit narrow genetic variation of commercial varieties and upgrade yield performance, e.g., in crops of wheat (Fasoula, 1990; Tokatlidis et al., 2006), maize (Tokatlidis, 2000), cotton (Tokatlidis et al., 2011b), barley (Ben Ghanem et al., 2018), and soybean (Fasoula and Boerma, 2007; De Almeida Lopes et al., 2020). Tokatlidis and Vlachostergios (2016) also suggested a conservation honeycomb breeding procedure for sustainable stewardship of the landrace diversity and continuous adaptation to an ever-changing environment.

Crop plasticity refers to the plant's ability to adapt and cope with changes in its environment (Rotili et al., 2021). Five main benefits from the productive ideotype analyzed in the first half of this study, plus those reported as additional benefits, indicate that the density-independent variety promises traits relative to compensation mechanisms against stresses. Density-independent varieties exist in soybean; soybean exhibits high-phenotypic plasticity, including the ability to alter its growth and yield components as a function of the number of individuals per area, thus maintaining constant productivity over a wide range of densities (Suhre et al., 2014; Junior et al., 2018). In wheat varieties, Fischer et al. (2019) described optimum density ranging from 30 up to above 100 plants/m^2^. Density-independent varieties are versatile to offer flexibility and plasticity to environmental diversity and secure over-season stability (Tokatlidis, 2017).

## Conclusion

Crop spacing *via* density-independent varieties is mandated in the future to meet several requirements against the yield gap in grain-producing crops, which are as follows: (i) address density-dependence and ensure optimal resource use inter-seasonally; (ii) cope with the acquired intra-crop variation and optimize the resource use; (iii) compensate for missing plants and promote stability; (iv) incorporate the multi-genotypic variety and counteract unpredictable stresses; (v) adopt the low-input agriculture to conserve natural resources and protect the environment. Breeding for the density-independent variety *via* improved plant yield efficiency should be conducted at the nil-competition regime to (i) cope with the confounding effects of competition in recognition of the targeted genotypes of the productive ideotype; (ii) maximize the phenotypic expression to obtain seed for extensive progeny testing and maximize the phenotypic differentiation to facilitate selection from the very early segregating generations; (iii) optimize heritability, thanks to moderated environmental variance and experimental designs that sample the spatial heterogeneity; (iv) apply high selection pressure focusing exclusively on top for plant yield efficiency genotypes; (v) avoid the risk of bias selection or loss of desired genotypes due to proximity to empty hills. Density-independent varieties are expected to be versatile to offer flexibility and plasticity to environmental diversity.

**Closing note**: Due to the rapid growth of molecular breeding in the last decades, conventional breeding has moved into the background. The article points out that conventional breeding must play a decisive role to meet today's hot challenges, such as crop sustainability and food security. Norman Borlaug used to say, “We can do another green revolution, by doing what we did before, with common sense” (Hesser, 2006). In the author's view, this study is a matter of common sense. Close cooperation between the two breeding branches would undoubtedly boost the outcome *via* valuable tools, like molecular assisted selection (MAS), quantitative trait loci maps (QTL), and Genomics.

## Author contributions

The author confirms being the sole contributor of this work and has approved it for publication.

## Funding

This work has been partially funded by the European Union and Greek national funds through the Operational Program Competitiveness, Entrepreneurship and Innovation, under the call Research—Create—Innovate (Project Code: T1EDK-00739).

## Conflict of interest

The author declares that the research was conducted in the absence of any commercial or financial relationships that could be construed as a potential conflict of interest.

## Publisher's note

All claims expressed in this article are solely those of the authors and do not necessarily represent those of their affiliated organizations, or those of the publisher, the editors and the reviewers. Any product that may be evaluated in this article, or claim that may be made by its manufacturer, is not guaranteed or endorsed by the publisher.

## References

[B1] Abraham B. Araya H. Berhe T. Edwards S. Gujja B. Khadka R. B.. (2014). The system of crop intensification: reports from the field on improving agricultural production, food security, and resilience to climate change for multiple crops. Agric. Food Secur. 3, 4. 10.1186/2048-7010-3-4

[B2] Abraham E. M. Fasoulas A. C. (2001). Comparative efficiency of three selection methods in *D. glomerata* L. and *A. cristatum* L. J. Agr. Sci. 137, 173–178. 10.1017/S0021859601001265

[B3] Adhikari P. Araya H. Aruna G. Balamatti A. Banerjee S. Baskaran P.. (2018). System of crop intensification for more productive, resource-conserving, climate-resilient, and sustainable agriculture: experience with diverse crops in varying agroecologies, Int. J. Agric. Sustain. 16, 1–28, 10.1080/14735903.2017.1402504

[B4] Adler P. B. Smull D. Beard K. H. Choi R. T. Furniss T. Kulmatiski A.. (2018). Competition and coexistence in plant communities: intraspecific competition is stronger than interspecific competition. Ecol. Lett. 21, 1319–1329. 10.1111/ele.13098 29938882

[B5] Altieri M. A. Nicholls C. I. Henao A. Lana M. A. (2015). Agroecology and the design of climate change-resilient farming systems. Agron. Sustain. Dev. 35, 869–890. 10.1007/s13593-015-0285-2

[B6] Amelong A. Hernández F. Novoa A. D. Borrás L. (2017). Maize stand density yield response of parental inbred lines and derived hybrids. Crop Sci. 57, 32–39. 10.2135/cropsci2016.02.0083

[B7] Anderson W. Johansen C. Siddique H. M. (2016). Addressing the yield gap in rainfed crops: a review. Agron. Sustain. Dev. 36, 18. 10.1007/s13593-015-0341-y

[B8] Anderson W. K. (1986). Some relationships between plant population, yield components and grain yield of wheat in a Mediterranean environment. Aust. J. Agric. Res. 37, 219–233. 10.1071/AR9860219

[B9] Anderson W. K. Sharma D. L. Shackley B. J. D'Antuono M. F. (2004). Rainfall, sowing time, soil type, and cultivar influence optimum plant population for wheat in Western Australia. Aust. J. Agric. Res. 55, 921–930. 10.1071/AR03248

[B10] Assefa Y. Carter P. Hinds M. Bhalla G. Schon R. Jeschke M.. (2018). Analysis of long term study indicates both agronomic optimal plant density and increase maize yield per plant contributed to yield gain. Sci. Rep. 8, 4937. 10.1038/s41598-018-23362-x 29563534 PMC5862987

[B11] Avdikos I. D. Tagiakas R. Mylonas I. Xynias I. N. Mavromatis A. G. (2021). Assessment of tomato recombinant lines in conventional and organic farming systems for productivity and fruit quality traits. Agronomy 11, 129. 10.3390/agronomy11010129

[B12] Bastos L. M. Carciochi W. Lollato R. P. Jaenisch B. R. Rezende C. R. Schwalbert R.. (2020). Winter wheat yield response to plant density as a function of yield environment and tillering potential: a review and field studies. Front. Plant Sci. 11, 54. 10.3389/fpls.2020.00054 PMC7066254 32194579

[B13] Batzios D. P. Roupakias D. G. Kechagia U. Galanopoulou-Sendouca S. (2001). Comparative efficiency of honeycomb and conventional pedigree methods of selection for yield and fiber quality in cotton (*Gossypium* spp.). Euphytica 122, 203–211. 10.1023/A:1012718715149

[B14] Ben Ghanem H. B. Najar A. Udupa S. Kumari S. G. Amri A. Rezgui S.. (2018). Exploiting intra-cultivar variation to select for *Barley yellow* dwarf virus-PAV (BYDV-PAV) resistance in barley. Can. J. Plant Sci. 98, 930–946. 10.1139/cjps-2017-0364

[B15] Benjamin L. R. (2017). Growth analysis, crops, in Encyclopedia of Applied Plant Sciences, 2nd Edn, eds Thomas B.., (Elsevier), 23–28. 10.1016/B978-0-12-394807-6.00225-2

[B16] Berzsenyi Z. Tokatlidis I. S. (2012). Density-dependence rather than maturity determines hybrid selection in dryland maize production. Agron. J. 104, 331–336. 10.2134/agronj2011.0205

[B17] Brabencová S. Ihnatová I. Potěšil D. Fojtová M. Fajkus J. Zdráhal Z.. (2017). Variations of histone modification patterns: contributions of inter-plant variability and technical factors. Front. Plant Sci. 8, 2084. 10.3389/fpls.2017.02084 PMC5725443 29270186

[B18] Brás T. A. Seixas J. Carvalhais N. Jägermeyr J. (2021). Severity of drought and heatwave crop losses tripled over the last five decades in Europe. Environ. Res. Lett. 16, 065012. 10.1088/1748-9326/abf004

[B19] Bussemakers A. Bos I. (1999). The effect of interplant distance on the effectiveness of honeycomb selection in spring rye. III. Accumulated results of five selection cycles. Euphytica 105, 229–237. 10.1023/A:1003418123013

[B20] Chapagain T. Good A. (2015). Yield and production gaps in rainfed wheat, barley, and canola in Alberta. Front. Plant Sci. 6, 90. 10.3389/fpls.2015.00990 26635824 PMC4646961

[B21] Chatzoglou T. Tokatlidis I. S. (2012). Short communication. Decision on germplasm choice to apply breeding within a local population of common vetch is affected by crowding. Span. J. Agric. Res. 10, 752–755. 10.5424/sjar/2012103-641-11

[B22] Chauhan S. Darvishzadeh R. van Delden S. H. Boschetti M. Nelson A. (2021). Mapping of wheat lodging susceptibility with synthetic aperture radar data. Remote Sens. Environ. 259, 112427. 10.1016/j.rse.2021.112427

[B23] Christakis P. A. Fasoulas A. C. (2002). The effects of the genotype by environmental interaction on the fixation of heterosis in tomato. J. Agric. Sci. 139, 55–60. 10.1017/S0021859602002198

[B24] Ciampitti I. A. Vyn T. J. (2014). Understanding global and historical nutrient use efficiencies for closing maize yield gaps. Agron. J. 106, 2107–2117. 10.2134/agronj14.0025

[B25] De Almeida Lopes K. B. Pípolo A. E. Bazzo J. H. B. Zucareli C. (2020). Intracultivar selection for seed quality of soybeans in an ultra-low-density selection model (honeycomb selection designs). Acta Sci. Agron. 42, e44299. 10.4025/actasciagron.v42i1.44299

[B26] Donald C. M. (1963). Competition among crop and pasture plants. Adv. Agron. 15, 1–118. 10.1016/S0065-2113(08)60397-1

[B27] Donald C. M. (1968). The breeding of crop ideotypes. Euphytica 17, 385–403. 10.1007/BF00056241

[B28] Donald C. M. (1981). Competitive plants, communal plants and yield in wheat crops, in Wheat Science: Today and Tomorrow, eds Evans L. T. Peacock W. J. (Cambridge: Cambridge University Press), 223–247.

[B29] Donald C. M. Hamblin J. (1976). The biological yield and harvest index of cereals as agronomic and plant breeding criteria. Adv. Agron. 28, 361–405. 10.1016/S0065-2113(08)60559-3

[B30] Döring T. F. Knapp S. Cohen J. E. (2015). Taylor's power law and the stability of crop yields. Field Crops Res. 183, 294–302. 10.1016/j.fcr.2015.08.005

[B31] Duvick D. N. (2005). The contribution of breeding to yield advances in maize (*Zea ma*ys L.). Adv. Agron. 86, 83–145. 10.1016/S0065-2113(05)86002-X

[B32] Eash L. Fonte S. J. Sonder K. Honsdorf N. Schmidt A. Govaerts B.. (2019). Factors contributing to maize and bean yield gaps in Central America vary with site and agroecological conditions. J. Agric. Sci. 157, 300–317. 10.1017/S0021859619000571

[B33] Echarte L. Luque S. Andrade F. H. Sandras V. O. Cirilo A. Otegui M. E.. (2000). Response of maize kernel number to plant population in Argentinean hybrids released between 1965 and 1993. Field Crops Res. 68, 1–8. 10.1016/S0378-4290(00)00101-5

[B34] Egli D. B. Hatfield J. L. (2014). Yield gaps and yield relationships in central US soybean production systems. Agron.J. 106, 560–566. 10.2134/agronj2013.0364

[B35] Falconer D. S. (1989). Introduction to Quantitative Genetics, 3rd Edn. New York, NY: Longman Scientific and Technical.

[B36] Fang Y. Xu B. Turner N. C. Li F. (2010). Grain yield, dry matter accumulation and remobilization, and root respiration in winter wheat as affected by seeding rate and root pruning. Eur. J. Agron. 33, 257–266. 10.1016/j.eja.2010.07.001

[B37] Farias M. Casa R. T. Gava F. Fiorentin O. A. Gonçalves M. J. Martins F. C. (2019). Effect of soybean plant density on stem blight incidence. Summa Phytopathol. 45, 247–251. 10.1590/0100-5405/188813

[B38] Fasoula D. A. (1990). Correlations between auto-, allo- and nilcompetition and their implications in plant breeding. Euphytica 50, 57–62. 10.1007/BF00023161

[B39] Fasoula D. A. Fasoula V. A. (1997). Competitive ability and plant breeding. Plant Breed. Rev. 14, 89–138. 10.1002/9780470650073.ch4

[B40] Fasoula D. A. Ioannides I. M. Omirou M. (2020). Phenotyping and plant breeding: overcoming the barriers. Front. Plant Sci. 10, 1713. 10.3389/fpls.2019.01713 31998353 PMC6962186

[B41] Fasoula V. A. (2013). Prognostic breeding: a new paradigm for crop improvement. Plant Breed. Rev. 37, 297–347. 10.1002/9781118497869.ch6

[B42] Fasoula V. A. Boerma H. R. (2007). Intra-cultivar variation for seed weight and other agronomic traits within three elite soybean cultivars. Crop Sci. 47, 367–373. 10.2135/cropsci2005.09.0334

[B43] Fasoula V. A. Fasoula D. A. (2000). Honeycomb breeding: principles and applications. Plant Breed. Rev. 18, 177–250. 10.1002/9780470650158.ch4

[B44] Fasoula V. A. Fasoula D. A. (2002). Principles underlying genetic improvement for high and stable crop yield potential. Field Crop Res. 75, 191–209. 10.1016/S0378-4290(02)00026-6

[B45] Fasoula V. A. Tokatlidis I. S. (2012). Development of crop cultivars by honeycomb breeding. Agron. Sustain. Dev. 32, 161–180. 10.1007/s13593-011-0034-0

[B46] Fasoula V. A. Tollenaar M. (2005). The impact of plant population density on crop yield and response to selection in maize. Maydica 50, 39–48.

[B47] Fasoulas A. (1981). Principles and Methods of Plant Breeding, Publ. 11. Thessaloniki: Department of Genetics and Plant Breeding, Aristotle University of Thessaloniki.

[B48] Fasoulas A. C. (1973). A New Approach to Breeding Superior Yielding Varieties, Pub. 3. Thessaloniki: Department of Genetics and Plant Breeding, Aristotle University of Thessaloniki.

[B49] Fasoulas A. C. (1987). A moving block evaluation technique for improving the efficiency of pedigree selection. Euphytica 36, 473–478. 10.1007/BF00041490

[B50] Fasoulas A. C. (1988). The Honeycomb Methodology of Plant Breeding. Thessaloniki: A.C. Fasoulas.

[B51] Fasoulas A. C. (1993). Principles of Crop Breeding. Thessaloniki: A.C. Fasoulas.

[B52] Fasoulas A. C. Fasoula V. A. (1995). Honeycomb selection designs. Plant Breed. Rev. 13, 87–139. 10.1002/9780470650059.ch3

[B53] Fasoulas A. C. Zaragotas D. A. (1990). New Developments in the Honeycomb Selection Designs, Pub. 12. Thessaloniki: Department of Genetics and Plant Breeding, Aristotle University of Thessaloniki.

[B54] Fischer R. A. (2020). Breeding wheat for increased potential yield: contrasting ideas from Donald and Fasoulas, and the case for early generation selection under nil competition. Field Crops Res. 252, 107782. 10.1016/j.fcr.2020.107782

[B55] Fischer R. A. Byerlee D. Edmeades G. O. (2009). Can technology deliver on the yield challenge to 2050?, in Proceedings of the Expert Meeting on How to Feed the World in 2050 (Rome: FAO), 24–26.

[B56] Fischer R. A. Kertesz Z. (1976). Harvest index in spaced plant populations and grain weight in microplots as indicators of yielding ability in spring wheat. Crop Sci. 16, 55–59. 10.2135/cropsci1976.0011183X001600010014x

[B57] Fischer R. A. Moreno Ramos O. H. Ortiz Monasterio I. Sayre K. D. (2019). Yield response to plant density, row spacing and raised beds in low latitude spring wheat with ample soil resources: an update. Field Crops Res. 232, 95–105. 10.1016/j.fcr.2018.12.011

[B58] Fischer R. A. Rebetzke G. J. (2018). Indirect selection for potential yield in early-generation, spaced plantings of wheat and other small-grained cereals: a review. Crop Pasture Sci. 69, 439–459. 10.1071/CP17409

[B59] Friedman S. P. (2016). Evaluating the role of water availability in determining the yield–plant population density relationship. Soil Sci. Soc. Am. J. 80, 563–578. 10.2136/sssaj2015.11.0395

[B60] Gaba S. Caneill J. Nicolardot B. Perronne R. Bretagnolle V. (2018). Crop competition in winter wheat has a higher potential than farming practices to regulate weeds. Ecosphere 9, e02413. 10.1002/ecs2.2413

[B61] Gireesh S. Kumbhare N. V. Nain M. S. Kumar P. Gurung B. (2019). Yield gap and constraints in production of major pulses in Madhya Pradesh and Maharashtra. Indian J. Agric. Res. 53, 104–107. 10.18805/IJARe.A-5067

[B62] Glotter M. Elliott J. (2016). Simulating US agriculture in a modern dust bowl drought. Nat. Plants 3, 16193. 10.1038/nplants.2016.193 27941818

[B63] Gonzalez V. H. Tollenaar M. Bowman A. Good B. Lee E. A. (2018). Maize yield potential and density tolerance. Crop Sci. 58, 472–485. 10.2135/cropsci2016.06.0547

[B64] Hamblin J. Knight R. Atkinson M. J. (1978). The influence of systematic micro-environmental variation on individual plant yield within selection plots. Euphytica 27, 497–503. 10.1007/BF00043175

[B65] Hamman B. Egli D. B. Koning G. (2002). Seed vigor, soilborne pathogens, preemergent growth, and soybean seedling emergence. Crop Sci. 42, 451–457. 10.2135/cropsci2002.4510

[B66] Hansen K. A. Martin J. M. Lanning S. P. Talbert L. E. (2005). Correlation of genotype performance for agronomic and physiological traits in space-planted vs. densely seeded conditions. Crop Sci. 45, 1023–1028. 10.2135/cropsci2004.0194

[B67] Hashemi-Dezfouli A. Herbert S. J. (1992). Intensifying plant population response of corn with artificial shade. Agron. J. 84, 547–551. 10.2134/agronj1992.00021962008400040001x

[B68] Hesser L. (2006). The Man Who Fed the World: Nobel Peace Prize Laureate Norman Borlaug and his Battle to End World Hunger. An Authorized Biography, 1st Edn. Dallas, TX: Durban House Publishing Company, Inc. USA.

[B69] Isbell F. (2015). Agroecology: agroecosystem diversification. Nat. Plants 1, 15041. 10.1038/nplants.2015.41 27247041

[B70] Joernsgaard B. Halmoe S. (2002). Intra-field yield variation over crops and years. Eur. J. Agron. 19, 23–33. 10.1016/S1161-0301(02)00016-3

[B71] Junior A. A. B. de Oliveira M. C. N. Julio Cezar Franchini J. C. Debiasi H. Zucareli C. Sampaio Ferreira A. S.. (2018). Phenotypic plasticity in a soybean cultivar with indeterminate growth type. Pesq. Agropec. Bras. 53, 1038–1044. 10.1590/s0100-204x2018000900007

[B72] Jurke C. J. Fernando W. G. D. (2008). Effects of seeding rate and plant density on sclerotinia stem rot incidence in canola. Arch. Phytopathol. Plant Prot. 41, 142–155, 10.1080/03235400600679743

[B73] Kargiotidou A. Chatzivassiliou E. Sinapidou E. Papageorgiou A. Skaracis G. Tokatlidis I. S. (2014). Selection at ultra-low density highlights plants escaping virus infection and leads towards high-performing pure-line cultivars in lentil. J. Agric. Sci. 152, 749–758. 10.1017/S0021859613000403

[B74] Kargiotidou A. Vlachostergios D. N. Tzantarmas C. Mylonas I. Foti C. Menexes G.. (2016). Addressing huge spatial heterogeneity induced by virus infections in lentil breeding trials. J. Biol. Res. Thessalon. 23, 2. 10.1186/s40709-016-0039-6 26933651 PMC4772466

[B75] Kotzamanidis S. T. Lithourgidis A. S. Roupakias D. G. (2009). Short communication. Plant density effect on the individual plant to plant yield variability expressed as coefficient of variation in barley. Span. J. Agric. Res. 7, 607–610. 10.5424/sjar/2009073-457

[B76] Kristoffersen R. Jørgensen L. N. Eriksen L. B. Nielsen G. C. Kiær P. L. (2020). Control of Septoria tritici blotch by winter wheat cultivar mixtures: meta-analysis of 19 years of cultivar trials. Field Crops Res. 249, 107696. 10.1016/j.fcr.2019.107696

[B77] Kyriakou D. T. Fasoulas A. C. (1985). Effects of competition and selection pressure on yield response in winter rye (*Secale cereale* L.). Euphytica 34, 883–895. 10.1007/BF00035428

[B78] Laker M. C. Nortjé G. P. (2019). Review of existing knowledge on soil crusting in South Africa. Adv. Agron. 155, 189–242. 10.1016/bs.agron.2019.01.002

[B79] Lal R. (2003). Soil erosion and the global carbon budget. Environ. Int. 29, 437–450. 10.1016/S0160-4120(02)00192-7 12705941

[B80] Lamichhane J.R Debaeke P. Steinberg C. You M.P.. (2018). Abiotic and biotic factors affecting crop seed germination and seedling emergence: a conceptual framework. Plant Soil 432, 1–28. 10.1007/s11104-018-3780-9

[B81] Lavalle C. Micale F. Houston T. D. Camia A. Hiederer R. Lazar C.. (2009). Climate change in Europe. 3. Impact on agriculture and forestry. A review. Agron. Sustain. Dev. 29, 433–446. 10.1051/agro/200806834427014

[B82] Lichtfouse E. Navarrete M. Debaeke P. Souchére V. Alberola C. Ménassieu J. (2009). Agronomy for sustainable agriculture: a review. Agron. Sustain. Dev. 29, 1–6. 10.1051/agro:2008054

[B83] Liu W. Tollenaar M. (2009). Response of yield heterosis to increasing plant density in maize. Crop Sci. 49, 1807–1816. 10.2135/cropsci2008.07.0422

[B84] Lollato R. P. Ruiz Diaz D. A. DeWolf E. Knapp M. Peterson D. E. Fritz A. K. (2019). Agronomic practices for reducing wheat yield gaps: a quantitative appraisal of progressive producers. Crop Sci. 59, 333. 10.2135/cropsci2018.04.0249

[B85] Lopes M. S. Araus J. L. van Heerden P. D. R. Foyer C. H. (2011). Enhancing drought tolerance in C4 crops. J. Exp. Bot. 62, 3135–3153. 10.1093/jxb/err105 21511912

[B86] Lungu D. M. Kaltsikes P. J. Larter E. N. (1987). Honeycomb selection for yield in early generation in spring wheat. Euphytica 36, 831–839. 10.1007/BF00051867

[B87] Maddonni G. A. Otegui M. E. (2004). Intra-specific competition in maize: early establishment of hierarchies among plants affects final kernel set. Field Crops Res. 85, 1–13. 10.1016/S0378-4290(03)00104-7

[B88] Mansfield B. D. Mumm R. H. (2014). Survey of plant density tolerance in U. S. maize germplasm. Crop Sci. 54, 157–173. 10.2135/cropsci2013.04.0252

[B89] Maximillian J. Brusseau M. L. Glenn E. P. Matthias A. D. (2019). Pollution and environmental perturbations in the global system. Environ. Pollut. Sci. 457–476. 10.1016/B978-0-12-814719-1.00025-2

[B90] Mayer L. I. Rossini M. A. Maddonni G. A. (2012). Inter-plant variation of grain yield components and kernel composition of maize crops grown under contrasting nitrogen supply. Field Crops Res. 125, 98–108. 10.1016/j.fcr.2011.09.004

[B91] Mirás-Avalos J. M. Baveye P. C. (2018). Editorial: agroecosystems facing global climate change: the search for sustainability. Front. Env. Sci. 6, 135. 10.3389/fenvs.2018.00135

[B92] Missaoui A. M. Fasoula V. A Bouton J. H. (2005). The effect of low plant density on response to selection for biomass production in switchgrass. Euphytica 142, 1–12. 10.1007/s10681-005-0149-y

[B93] Mitchell J. W. Baker R. J. Knott D. R. (1982). Evaluation of honeycomb selection for single plant yield in durum wheat. Crop Sci. 22, 840–843. 10.2135/cropsci1982.0011183X002200040033x

[B94] Mondo V. H. V. Cicero S. M. Dourado-Neto D. Pupim T. L. Dias M. A. N. (2013). Effect of seed vigor on intraspecific competition and grain yield in maize. Agron. J. 105, 222–228. 10.2134/agronj2012.0261

[B95] Mundt C. C. (2002). Use of multiline cultivars and cultivar mixtures for disease management. Annu. Rev. Phytopathol. 40, 381–410. 10.1146/annurev.phyto.40.011402.113723 12147765

[B96] Mylonas I. Sinapidou E. Remountakis E. Sistanis I. Pankou C. Ninou E.. (2020). Improved plant yield efficiency alleviates the erratic optimum density in maize. Agron. J. 112, 1690–1701. 10.1002/agj2.20187

[B97] Ninou E. G. Mylonas I. G. Tsivelikas A. Ralli P. Dordas C. Tokatlidis I. S. (2014). Wheat landraces are better qualified as potential gene pools at ultraspaced rather than densely grown conditions. Sci. World J. 2014, 957472. 10.1155/2014/957472 24955427 PMC4037628

[B98] Ntanos D. A. Roupakias D. G. (2001). Comparative efficiency of two breeding methods for yield and quality in rice. Crop Sci. 41, 345–350. 10.2135/cropsci2001.412345x

[B99] O'Brien P. Kral-O'Brien K. Hatfield J. L. (2021). Agronomic approach to understanding climate change and food security. Agron. J. 2021, 1–11. 10.1002/agj2.20693

[B100] Ohtsuki A. Sasaki A. (2006). Epidemiology and disease-control under gene-for-gene plant–pathogen interaction. J. Theor. Biol. 238, 780–794. 10.1016/j.jtbi.2005.06.030 16085107

[B101] Olsen J. Weiner J. (2007). The influence of *Triticum aestivum* density, sowing pattern and nitrogen fertilization on leaf area index and its spatial variation. Basic Appl. Ecol. 8, 252–257. 10.1016/j.baae.2006.03.013

[B102] Omer C. Nisan Z. Rav-David D. Elad Y. (2021). Effects of agronomic practices on the severity of sweet basil downy mildew (*Peronospora belbahrii*). Plants 10, 907. 10.3390/plants10050907 PMC8147145 33946467

[B103] Pan X. Y. Wang G. X. Yang H. M. Wei X. P. (2003). Effect of water deficits on within-plot variability in growth and grain yield of spring wheat in northwest China. Field Crops Res. 80, 195–205. 10.1016/S0378-4290(02)00175-2

[B104] Pankou C. Koulymboudi L. Papathanasiou F. Gekas F. Papadopoulos I. Sinapidou E. (in press). Testing taylor's power law association of maize interplant variation with mean grain yield. J. Integr. Agric. 10.1016/j.jia.2022.08.103.

[B105] Papadakis J. (1935a). Varieties experiments in pots. Thessaloniki PI. Breed. Inst. Sci. Bull. 20, 26.

[B106] Papadakis J. (1935b). The pocket method of varieties experiments. Thessaloniki PI. Breed. Inst. Sci. Bull. 21, 8.

[B107] Papadakis J. (1937a). Est-ce Seulement d'apres le Rendement en grain que se fait la Sclection Naturelle chez les Plantes Cultivees? Thessaloniki Pl. Breed. Inst. Sci. Bull. 26.

[B108] Papadakis J. (1940a). Comparaison de différentes méthodes d'expérimentation phytotechnique. Rev. Agr. Agron. 7, 297–362.

[B109] Papadakis J. (1940b). The relation of the number of tillers per unit area to the yield of wheat and its bearing on fertilizing and breeding this plant-the space factor. Soil Sci. 50, 369–388.

[B110] Papadakis J. (1941). An important effect of soil colloids on plant growth. Soil Sci. 52, 283–290.

[B111] Papadakis J. (1982). Errores en la ciencia de nuestros días. Más especialmente en ciencia del suelo, ecologia y agronomía. Pédol XIX, 91–104.

[B112] Papadakis,. J. (1937b). Expériences et Perfectionnements à la Méthode des Pocquets pour Essais de Variétés. Thessaloniki PI. Breed. Inst. Sci. Bull. 27, 32.

[B113] Pasini R. J. Bos I. (1990). The effect of interplant distance on the effectiveness of honeycomb selection. II Results of the second selection cycle. Euphytica 50, 147–153. 10.1007/BF00023638

[B114] Pommel B. Bonhomme R. (1998). Variations in the vegetative and reproductive systems in individual plants of an heterogeneous maize crop. Eur. J. Agron. 8, 39–49. 10.1016/S1161-0301(97)00012-9

[B115] Rebetzke G. J. Fischer R. A. van Herwaarden A. F. Bonnett D. G. Chenu K. Rattey A. R.. (2014). Plot size matters: interference from intergenotypic competition in plant phenotyping studies. Funct. Plant Biol. 41, 107–118. 10.1071/FP13177 32480971

[B116] Reynolds M. P. Acevedo E. Sayre K. D. Fischer R. A. (1994). Yield potential in modern wheat varieties: it association with a less competitive ideotype. Field Crops Res. 37, 149–160. 10.1016/0378-4290(94)90094-9

[B117] Rosielle A. A. Hamblin J. (1981). Theoretical aspects of selection for yield in stress and non-stress environments. Crop Sci. 21, 943–946. 10.2135/cropsci1981.0011183X002100060033x

[B118] Rossini M. A. Maddonni G. A. Otegui M. E. (2016). Multiple abiotic stresses on maize grain yield determination: additive vs. multiplicative effects. Field Crops Res. 121, 373–380. 10.1016/j.fcr.2011.01.003

[B119] Rotili D. H. Sadras V. O. GabrielaAbeledo L. G. Ferreyra J. M. Micheloud J. R. Duarte G.. (2021). Impacts of vegetative and reproductive plasticity associated with tillering in maize crops in low-yielding environments: a physiological framework. Field Crops Res. 265, 108107. 10.1016/j.fcr.2021.108107

[B120] Rufo M. L. Gentry L. F. Henninger A. S. Seebauer J. R. Below F. E. (2015). Evaluating management factor contributions to reduce corn yield gaps. Agron. J. 107, 495–505. 10.2134/agronj14.0355

[B121] Sadras V. O. Trápani N. Pereyra V. R. López Pereira M. Quiroz F. Mortarini M. (2000). Intraspecific competition and fungal diseases as sources of variation in sunflower yield. Field Crops Res. 67, 51–58. 10.1016/S0378-4290(00)00083-6

[B122] Schwinning S. Weiner J. (1998). Mechanisms determining the degree of size asymmetry in competition among plants. Oecologia 113, 447–455. 10.1007/s004420050397 28308024

[B123] Sedgley R. H. (1991). An appraisal of the donald ideotype after 21 years. Field Crops Res. 26, 93–112. 10.1016/0378-4290(91)90031-P

[B124] Senapati N. Semenov M. A. (2020). Large genetic yield potential and genetic yield gap estimated for wheat in Europe. Global Food Sec. 24, 100340. 10.1016/j.gfs.2019.100340 32190539 PMC7063691

[B125] Shao H. Shi D. Shi W. Ban X. Chen Y. Ren W.. (2019). Genotypic difference in the plasticity of root system architecture of field-grown maize in response to plant density. Plant Soil 439, 201–217. 10.1007/s11104-019-03964-8

[B126] Siddique K. H. M. Sedgley R. H. Marshall C. (1984). Effect of plant density on growth and harvest index of branches in chickpea (*Cicer arietinum* L.). Field Crops Res. 9, 193–203. 10.1016/0378-4290(84)90025-X

[B127] Smutná P. Tokatlidis I. S. (2021). Testing Taylor's power law association of winter wheat variation with mean yield at two contrasting soils. Eur. J. Agron. 26, 126268. 10.1016/j.eja.2021.126268

[B128] Spink J. H. Semere T. Sparkes D. L. Whaley J. M. Foulkes M. J. Clare R. W.. (2000). Effect of sowing date on optimum plant density of winter wheat. Ann. Appl. Biol. 137, 179–188. 10.1111/j.1744-7348.2000.tb00049.x

[B129] Stafford J. V. Ambler B. Lark R. M. Catt J. (1996). Mapping and interpreting the yield variation in cereal crops. Comput. Elect. Agric. 14, 101–119. 10.1016/0168-1699(95)00042-9

[B130] Suhre J. J. Weidenbenner N. H. Rowntree S. C. Wilson E. W. Naeve S. L. Conley S. P.. (2014). Soybean yield partitioning changes revealed by genetic gain and seeding rate interactions. Agron. J. 106, 1631–1642. 10.2134/agronj14.0003

[B131] Tokatlidis I. Vlachostergios D. (2016). Sustainable stewardship of the landrace diversity. Diversity 8, 29. 10.3390/d8040029

[B132] Tokatlidis I. S. (2000). Variation within maize lines and hybrids in the absence of competition and relation between hybrid potential yield per plant with line traits. J. Agric. Sci. 134, 391–398. 10.1017/S0021859699007637

[B133] Tokatlidis I. S. (2013). Adapting maize crop to climate change. Agron. Sustain. Dev. 33, 63–79. 10.1007/s13593-012-0108-7

[B134] Tokatlidis I. S. (2014). Addressing the yield by density interaction is a prerequisite to bridge the yield gap of rainfed wheat. Ann. Appl. Biol. 165, 27–42. 10.1111/aab.12121

[B135] Tokatlidis I. S. (2016). Sampling the spatial heterogeneity of the honeycomb model in maize and wheat breeding trials: analysis of secondary data compared to popular classical designs. Exp. Agric. 52, 371–390. 10.1017/S0014479715000150

[B136] Tokatlidis I. S. (2017). Crop adaptation to density to optimise grain yield: breeding implications. Euphytica 213, 92. 10.1007/s10681-017-1874-8

[B137] Tokatlidis I. S. Dordas C. Papathanasiou F. Papadopoulos I. Pankou C. Gekas F.. (2015). Improved plant yield efficiency is essential for maize rainfed production. Agron. J. 107, 1011–1018. 10.2134/agronj14.059923671071

[B138] Tokatlidis I. S. Has V. Melidis V. Has I. Mylonas I. Evgenidis G.. (2011a). Maize hybrids less dependent on high plant densities improve resource use efficiency in rainfed and irrigated conditions. Field Crops Res. 120, 345–351. 10.1016/j.fcr.2010.11.006

[B139] Tokatlidis I. S. Has V. Mylonas I. Has I. Evgenidis G. Melidis V.. (2010a). Density effects on environmental variance and expected response to selection in maize (*Zea mays* L.). Euphytica 174, 283–291. 10.1007/s10681-010-0160-924424414

[B140] Tokatlidis I. S. Koutroubas S. D. (2004). A review study of the maize hybrids' dependence on high plant populations and its implications on crop yield stability. Field Crops Res. 88, 103–114. 10.1016/j.fcr.2003.11.013

[B141] Tokatlidis I. S. Koutsika-Sotiriou M. Fasoulas A. C. (2001). The development of density independent maize hybrids. Maydica 46, 21–25.

[B142] Tokatlidis I. S. Koutsika-Sotiriou M. Fasoulas A. C. Tsaftaris A. S. (1998). Improving maize hybrids for potential yield per plant. Maydica 43, 123–129.

[B143] Tokatlidis I. S. Koutsika-Sotiriou M. Tamoutsidis E. (2005). Benefits from using maize density-independent hybrids. Maydica 50, 9–17.

[B144] Tokatlidis I. S. Papadopoulos I. I. Baxevanos D. Koutita O. (2010b). GxE effects on single-plant selection at low density for yield and stability in climbing dry bean. Crop Sci. 50, 775–783. 10.2135/cropsci2009.08.0459

[B145] Tokatlidis I. S. Remountakis E. (2020). The Impacts of interplant variation on aboveground biomass, grain yield, and harvest index in maize. Int. J. Plant Prod. 14, 57–65. 10.1007/s42106-019-00067-3

[B146] Tokatlidis I. S. Tsikrikoni C. Lithourgidis A. S. Tsialtas J. T. Tzantarmas C. (2011b). Intra-cultivar variation in cotton: response to single-plant yield selection at low density. J. Agric. Sci. 149, 197–204. 10.1017/S0021859610000596

[B147] Tokatlidis I. S. Xynias I. N. Tsialtas J. T. Papadopoulos I. I. (2006). Single-plant selection at ultra-low density to improve stability of a bread wheat cultivar. Crop Sci. 46, 90–97. 10.2135/cropsci2005.0125

[B148] Tollenaar M. Wu J. (1999). Yield improvement in temperate maize is attributable to greater stress tolerance. Crop Sci. 39, 1597–1604. 10.2135/cropsci1999.3961597x

[B149] Traka-Mavrona E. Georgakis D. Koutsika-Sotiriou M. Pritsa T. (2000). An integrated approach of breeding and maintaining an elite cultivar of snap bean. Agron. J. 92, 1020–1026. 10.2134/agronj2000.9251020x

[B150] Tsivelikas A. L. Ben Ghanem H. El-Baouchi A. Zakaria K. (2022). Single-plant selection at ultra-low density enhances buffering capacity of barley varieties and landraces to unpredictable environments and improves their agronomic performance. Front. Plant Sci. 13, 838536. 10.3389/fpls.2022.838536 35251108 PMC8895306

[B151] Uphoff N. Fasoula V. Iswandi A. Kassam A. Thakur A. K. (2015). Improving the phenotypic expression of rice genotypes: rethinking “intensification” for production systems and selection practices for rice breeding. Crop J. 3, 174–189. 10.1016/j.cj.2015.04.001

[B152] Van der Meulen A. Chauhan B. S. (2017). A review of weed management in wheat using crop competition. Crop Protect. 95, 38–44. 10.1016/j.cropro.2016.08.00428766148

[B153] Van Ittersum M. K. Cassman K. G. Grassini P. Wolf J. Tittonell P. Hochman Z. (2013). Yield gap analysis with local to global relevance: a review. Field Crops Res. 143, 4–17. 10.1016/j.fcr.2012.09.009

[B154] Vlachostergios D. N. Tzantarmas C. Kargiotidou A. Ninou E. Pankou C. Gaintatzi C.. (2018). Single-plant selection within lentil landraces at ultra-low density: a short-time tool to breed high yielding and stable varieties across divergent environments. Euphytica 214, 58. 10.1007/s10681-018-2139-x

[B155] Walsh M. J. (2019). Enhanced wheat competition effects on the growth, seed production, and seed retention of major weeds of Australian cropping systems. Weed Sci. 67, 657–665. 10.1017/wsc.2019.53

[B156] Weiner J. Du Y.-L. Zhang C. Qin X.-L.-L. Li F.-M.-M. (2017). Evolutionary agroecology: individual fitness and population yield in wheat (*Triticum aestivum*). Ecology 98, 2261–2266. 10.1002/ecy.1934 28783218

[B157] Wezel A. Soboksa G. McClelland S. Delespesse F. Boissau A. (2015). The blurred boundaries of ecological, sustainable, and agroecological intensification: a review. Agron. Sustain. Dev. 35, 1283–1295. 10.1007/s13593-015-0333-y

[B158] Whaley J. M. Kirby E. J. M. Spink J. H. Foulkes M. J. Sparkes D. L. (2004). Frost damage to winter wheat in the UK: the effect of plant population density. Eur. J. Agron. 21, 105–115. 10.1016/S1161-0301(03)00090-X

[B159] Wood G. A. Welsh J. P. Godwin R. J. Taylor J. C. Earl R. Knight S. M. (2003). Real-time measures of canopy size as a basis for spatially varying nitrogen applications to winter wheat sown at different seed rates. Biosyst. Eng. 84, 513–531. 10.1016/S1537-5110(03)00006-0

[B160] Yang W. Peng S. Laza R. C. Visperas R. M. Dionisio-Sese M. (2008). Yield gap analysis between dry and wet season rice crop grown under high-yielding management conditions. Agron. J. 100, 1390–1395. 10.2134/agronj2007.0356

[B161] Zeven A. C. (2002). Traditional maintenance breeding of landraces: 2. Practical and theoretical considerations on maintenance of variation of landraces by farmers and gardeners. Euphytica 123, 147–158. 10.1023/A:1014940623838

[B162] Zhai L. Xie R. Ming B. Li S. Ma D. (2018). Evaluation and analysis of intraspecific competition in maize: a case study on plant density experiment. J. Integr. Agr. 17, 2235–2244. 10.1016/S2095-3119(18)61917-3

[B163] Zhang X.-F. Luo C.-L. Mo F. Ren H.-X. Mburu D. Kavagi L.. (2021). Density-dependent maize (*Zea mays* L.) yield increase in trade-off in reproductive allocation and water use under ridge-furrow plastic-mulching. Field Crops Res. 264, 108102. 10.1016/j.fcr.2021.108102

